# On the Nature of the Transition State Characterizing Gated Molecular Encapsulations

**DOI:** 10.3390/molecules190914292

**Published:** 2014-09-11

**Authors:** Xiaoyong Lu, Bao-Yu Wang, Shigui Chen, Jovica D. Badjić

**Affiliations:** Department of Chemistry and Biochemistry, The Ohio State University, 100 West 18th Avenue, Columbus, OH 43210, USA

**Keywords:** molecular encapsulation, molecular gating, host-guest chemistry, liner free energy relationships, encapsulation mechanisms

## Abstract

Gated molecular encapsulations, with baskets of type **1**, are postulated to occur by the mechanism in which solvent molecule penetrates the inner space of **1**, through one of its apertures, while the residing guest simultaneously departs the cavity. In the transition state of the exchange, three pyridine-based gates are proposed to assume an open position with both incoming solvent and departing guest molecules interacting with the concave surface of the host. The More O’Ferrall-Jencks diagram and linear free energy relationships (LFERs) suggest a more advanced departure of the guest when bigger solvents partake in the displacement.

## 1. Introduction

The process of molecular encapsulation occurs, in concave hosts, via slippage, gating or full dissociation of self-assembled components [1]. Indeed, elucidating the mechanism of the operation of cavitands [2,3,4,5] has been an interest for controlling the outcome of chemical reactions [6], stabilizing reactive intermediates [7], or delivering useful molecules [8,9]. Accordingly, for the complexation of metal cations with crown ethers, Schneider and Cox reported that the rates by which cations access (*k*_in_) the macrocycles are fast (approaching a diffusion-controlled limit) while their departure from the complex (*k*_out_) is much slower corresponding to the thermodynamic stability (∆*G*°) of the complex itself [10]. Importantly, the corresponding mechanistic study [11] indicated an early transition state for the entrapment, resembling reactants to account for the absence of the selectivity in complexation events. For gated molecular encapsulations [12], however, a conformational change in a gated host creates an aperture permitting in/out exchange of guests. The complexation is, for this reason, occurring at a slower rate with the corresponding higher activation barrier referred to as constrictive binding [13]. As a result, one could tune the kinetics of molecular encapsulation by controlling conformational changes of the host [14]. Furthermore, the “opening” of some dynamic hosts, hemicarcerans [15,16] or self-folding capsules [17], were argued to constitute the rate-determining step of encapsulation. In other gating situations [18,19], however, the guest entrance generates enough van der Waals strain so that the encapsulation rate law exhibits a kinetic dependence in the concentration of both host and guest [20]. To our knowledge, there has been no particular study [21] about the nature of transition states characterizing gated encapsulations, and this is the objective of our paper.

**Figure 1:**
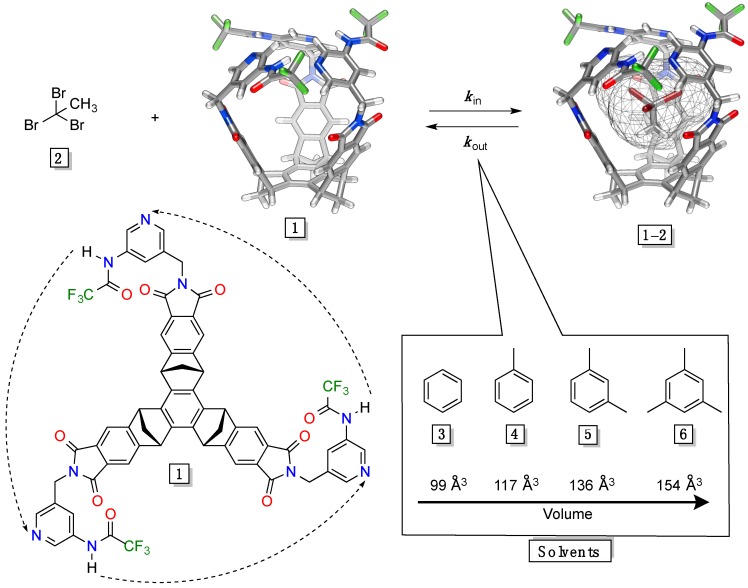
Energy-minimized (MMFFs, Spartan) and chemical structures of gated molecular basket **1** (V = 220 Å^3^). The kinetics of in/out trafficking of guest **2** (V = 107 Å^3^) to (*k*_in_) and from (*k*_out_) basket **1** was studied in solvents **3**–**6** (99–154 Å^3^).

Gated molecular baskets [22] have been designed and studied in our laboratory for almost a decade [23]. These hosts comprise a flat aromatic base that is fused to three bicycle[2.2.1]heptane rings forming a curved unit (V = 220 Å^3^, Figure 1).

Three phthalimides extend this structure into a bowl-shaped platform. Three aminopyridine rings [24], containing hydrogen-bonding donor and acceptor sites, are attached to the rim of the platform to, via CH_2_ “hinges”, act as gates that, by controlling the portal size, regulate the in/out encapsulation of small haloalkanes (V ~ 88–120 Å^3^) [25]. The formation of basket/guest complex was, in solvophobic CD_2_Cl_2_ solvent (V = 61 Å^3^) [26], found to be first- while the dissociation is zeroth-order in guests (Figure 1) [20]. In essence, the basket undergoes a rapid thermal racemization (∆*G*^‡^_rac_ < 11 kcal/mol) to, occasionally, permit the entrance/departure of guest molecules [27]. For a series of isosteric guests we found a linear dependence between the host-guest affinities (∆*G*°) and the free energies of activation (∆*G*^‡^*_in_* and ∆*G*^‡^*_out_*), which was fit to the following equation: ∆*G*^‡^*_in/out_* = ρ*_in/out_*∆*G*° + δ [28] On the basis of rather small ρ_in_ values (0.08–0.25), we hypothesized that an early transition state characterizes gated encapsulations [27,28]; this was additionally supported with a relatively poor stereo-selectivity of entrapments (*k_in_*^R^/*k_in_*^S^ ~ 2–3) [29]. *To investigate the nature of the transition state of gated molecular encapsulations in greater details*, we hereby used dynamic ^1^H-NMR spectroscopy to measure rate coefficients (*k*_out_) by which 1,1,1-tribromoethane **2** (V = 107 Å^3^) departs from the interior of basket **1** in four aromatic solvents **3**–**6** (Figure 1). In earlier studies [20], we surmised that the egress of guest molecule should be followed with the entrapment of solvent (Figure 1). It follows that the rate of such guest swap should change with increasingly bigger solvent molecules (99–154 Å^3^, Figure 1) via a steric imposition of the transition state of the transformation [21,30].

## 2. Results and Discussion

**Figure 2:**
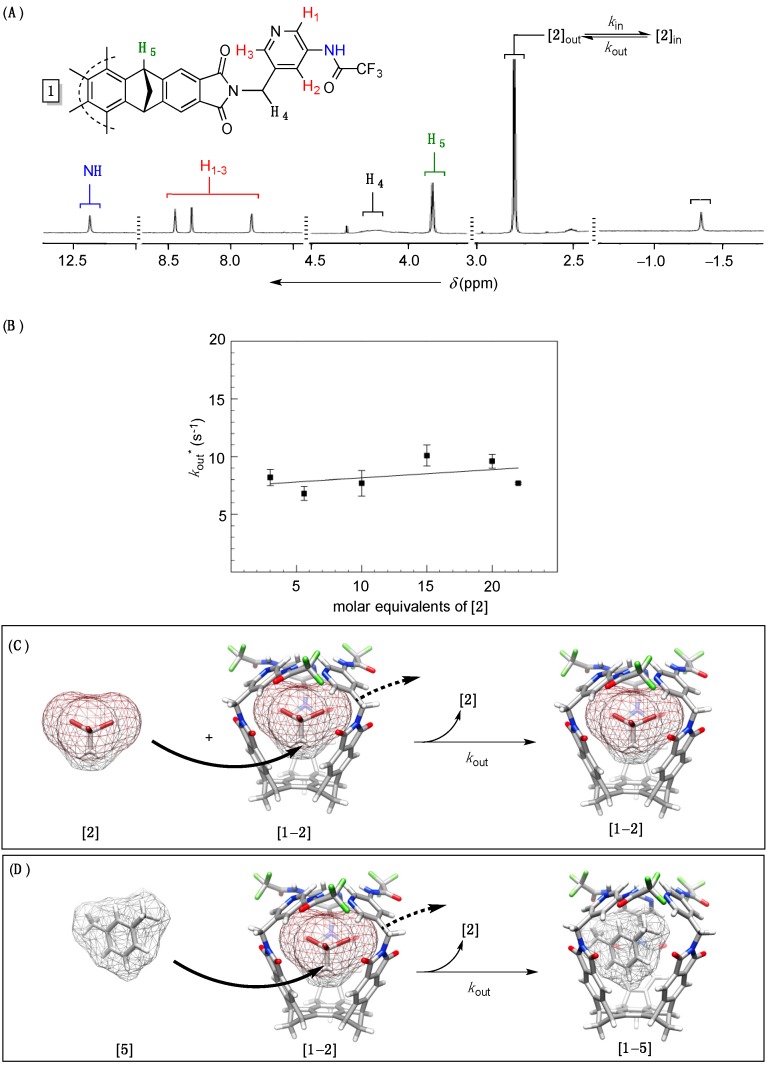
(**A**) ^1^H-NMR spectrum (400 MHz, 300.1 K) of *C*_3_ symmetric basket **1** (0.5 mg) suspended in *m*-xylene-d_10_ and containing guest **2** (6.5 mM); (**B**) A plot showing experimental rate coefficients *k*_out_ * (*s^−^*^1^), obtained from ^1^H,^1^H–EXSY NMR (400 MHz) measurements, as a function of the concentration of guest **2** with basket **1** (1.1 mg) in *m*-xylene-d_10_ at 300.1 K; The interchange ((**C**), MMFFs/Spartan) or solvent-displacement ((**D**), MMFFs/Spartan) mechanisms of the exchange).

**Figure 3:**
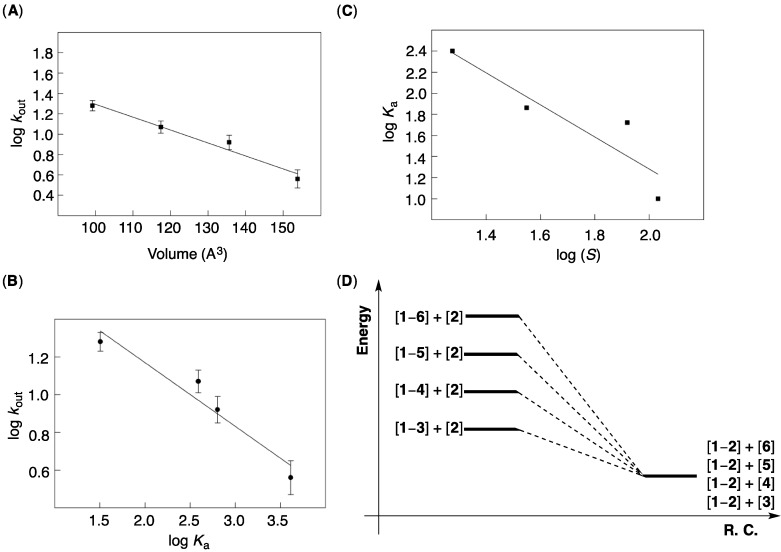
(**A**) A plot showing log *k*_out_ as function of van der Waals volume (Spartan) of solvents **3**–**6**. The data was fit to a linear function using the least-square method of analysis (R^2^ = 0.97, Sigma Plot); (**B**) A plot showing log *k*_out_ as function of the stability log *K*_a_ of [**1**–**2**] complex in solvents **3**–**6**. The data was fit to a linear function using the least-square method of analysis (*R*^2^ = 0.94, Sigma Plot); (**C**) A plot showing showing log *K*_a_ as function of the solubility log *S* of [**1**–**2**] complex in solvents **3**–**6**. The data was fit to a linear function using the least-square method of analysis (*R*^2^ = 0.94, Sigma Plot); (**D**) Reaction coordinate diagram showing the relative stability of [**1**–**solvent**] complexes.

Basket **1** (Figure 1) was prepared by following an optimized synthetic procedure [14]. It was poorly soluble in *m*-xylene-d_10_ (*vide supra*) forming a suspension at 298.0–348.0 K (Figure S1). Upon addition of 1,1,1-tribromoethane **2** (0.8–50 molar equivalents, Figure S4), however, the solid basket **1** was extracted into *m*-xylene-d_10_ to, perhaps, give a [**1**–**2**] complex. Indeed, the formation of [**1**–**2**] ensued on the basis of ^1^H-NMR spectroscopy and the intensity of signals corresponding to both host and guest (Figure 2A). DOSY NMR spectroscopic measurements (Figures S6–S9) were also in line with the formation of 1:1 complex in each solvent (*r*_H_ ~ 7–8 Å, Table 1) [31]; the experimental hydrodynamic radii correspond to the computed size (*d* ~ 14 Å, MMFFs) of [**1**–**2**] complex. Furthermore, two separate ^1^H- singlets corresponding to the guest inside the host (δ = −1.34 ppm, Figure 2A) and in bulk solvent (δ = 2.81 ppm, Figure 2A) suggested a slow in/out exchange of CH_3_CBr_3_ occurring at 300.1 K on the “time scale. Accordingly, we completed ^1^H,^1^H–EXSY NMR measurements (400 MHz, 300.1 K) to examine the kinetics characterizing the trafficking of complexed guest [**2**]_in_ into bulk solvent [**2**]_out_ (*k*_out_, Figure 2A); note that due to the nature of EXSY experiment [32], the experimentally determined rate coefficient *k*_out_ * is pseudo first-order in character (s^−1^, Table 1) and in line with the rate *v*_out_ * = *k*_out_ * [**1**–**2**]. On the basis of the reaction’s stoichiometry, however, the reaction’s rate could be *v*_out_ = *k*_out_ [**1**–**2**] (Figure 2A) and it follows that *k*_out_ * = *k*_out_ as long as the proposed model is valid. Since greater concentrations of guest [**2**] in solution (3.0–22.0 molar equivalents) had no measurable effects on the experimental rate constant *k*_out_ * (Figure 2B) we conclude that the process of decomplexation is zeroth-order in 1,1,1-tribromoethane **2** [20]. This, in turn, corroborates the absence of interchange mechanism (Figure 2C) [19], whereby free guests substitute the one of the same kind trapped in the host. Alternatively, a molecule of solvent **5** could approach [**1**–**2**] to via its sizeable side aperture “push” the encapsulated guest out and thereby give complex [**1**–**5**] (Figure 2D); note that, in this situation [28], the gates ought to “open” so that the substitution takes place. In line with this mechanistic hypothesis, varying the size of solvent molecules should have an effect on the rate by which guest **2** departs the basket: more sizeable compounds should create a greater van der Waals strain, while entering or exiting the host (vide infra), to affect the in/out exchange! Accordingly, we measured (^1^H,^1^H–EXSY 300.1 K) rate coefficients *k*_out_ corresponding to 1,1,1-tribromoethane **2** departing from [**1**–**2**] complex in three additional solvents: Benzene-d_6_ (**3**), toluene-d_8_ (**4**) and mesitylene-d_12_ (**6**). The rate coefficients *k*_out_ were, importantly, found to increase (while *k*_in_ decreased) for smaller solvents (Table 1). In fact, there exists a linear free energy relationship (LFER) with log *k*_out_ being proportional to the volume of solvent molecules (*R*^2^ = 0.97, Figure 3A).

To additionally probe the nature of the encapsulation events at hand, we used ^1^H-NMR spectroscopy to quantify the affinity (*K*_a_, M^−1^) of basket **1** for trapping **2** in each solvent (Table 1). The binding was found to be strongest in “large” mesitylene-d_12_ (V = 154 Å^3^) while weakest in “small” benzene-d_6_ (V = 99 Å^3^) [26]. The measured affinity constants (*K*_a_, Table 1) suggest for the stability of [**1**–**solvent**] complexes to be in the order: [**1**–**6**] < [**1**–**5**] < [**1**–**4**] < [**1**–**3**] (Figure 3D). In other words, the stability of [**1**–**2**] could be comparable in solvents **3**–**6**, having ε ~ 2–4 (Figure 3D) [33]. To further investigate this point of view, we used ^19^F-NMR spectroscopy (with C_6_F_6_ as internal standard, Figure S14) to quantify the solubility (*S*, Table 1) of basket **1** in solvents **3**–**6**. The solubility delineates a dynamic equilibrium between basket **1** in its solid state (always the same in any solvent) and as solute in solution and is thus proportional to the stability of [**1**–**solvent**] complexes. As log (*S*) commensurates with log *K*_a_ (Figure 3C), we conclude that in these two experiments the stability of [**1**–**solvent**] complexes primarily determines the state of each equilibrium. Consequently, the stability of [**1**–**2**] complex is, in aromatic solvents **3**–**6**, comparable and therefore shown in Figure 3D as one state. At last, the experimental order of stability of [**1**–**solvent**] complexes tracks an increased population of the inner space of basket **1** (approximated *PCs* are given in Table 1) [26].

**Table 1:** Diffusion coefficients (*D*) of [**1**–**2**] complex were obtained from DOSY NMR (400 MHz) at 300.1 K and converted into hydrodynamic radii (*r*_H_) using the Stokes-Einstein equation. The exchange rate constants *k*_out_ (*s^−^*^1^) were obtained from ^1^H,^1^H–EXSY NMR (300.1 K) and are shown as mean ± standard deviation (>10 measurements). The equilibrium constants *K*_a_ (M*^−^*^1^) were estimated from ^1^H NMR spectroscopic measurements (via signal integration) at 300.1 K, while *k*_in_ was calculated as *k*_in_ = *K*_a_ *k*_out_. Solubility (*S*) of basket **1**, in **3**–**6**, was determined with the assistance of ^19^F NMR spectroscopy at 300.1 K. Packing coefficients (PC) were calculated as *PC* (%) = *V*_guest_/*V*_host’s inner space_ [26].

	C_6_D_6_ (3)	C_6_D_5_CD_3_ (4)	C_6_D_4_ (CD_3_)_2_ (5)	C_6_D_3_ (CD_3_)_3_ (6)
*D* (10^−10^ m^2^s^−1^)	4.7 ± 0.1	5.9 ± 0.2	4.47 ± 0.06	4.21 ± 0.03
*r*_H_ (Å)	7.7	6.7	8.5	8.0
*k*_out_ (s^−1^)	19.2 ± 2.2	11.8 ± 1.6	8.3 ± 1.3	3.6 ± 0.8
*K*_a_ (M^−1^)	32	390	637	4123
*k_in_* (M^−1^s^−1^)	[6.1 ± 0.7] × 10^2^	[4.6 ± 0.6] × 10^3^	[5.3 ± 0.8] × 10^3^	[1.5 ± 0.3] × 10^4^
*S* (µmol/dm^3^)	108 ± 11	83 ± 8	35 ± 4	19 ± 2
*PC* (%)	45	53	62	70

**Figure 4:**
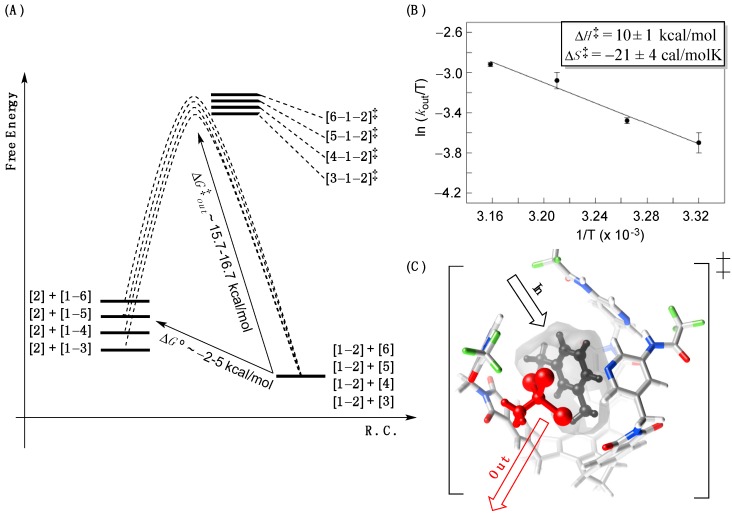
(**A**) A reaction coordinate diagram depicting kinetic and thermodynamic characteristics of the encapsulation; (**B**) The Eyring plot showing log *k*_out_/T as function of 1/T corresponding to the decomplexation of [**1**–**2**] complex in *m*-xylene-d_10_. The data was fit to a linear function using the least-square method of analysis (*R*^2^ = 0.98, SigmaPlot); (**C**) The energy minimized [**5**–**1**–**2**]^‡^ complex (MMFFs, Spartan) showing *m*-xylene-d_10_ entering while 1,1,1-tribromoethane **2** exiting the basket’s inner space.

Evidently, more favorable decomplexations of [**1**–**2**] (smaller log *K*_a_, Figure 3B) are, in solvents **3**–**6**, occurring at a faster rate (bigger log *k*_out_). The slope of the linear log *K*_a_/log *k_out_* function (Figure 3B) is equal to 0.3 (Figure 3B), with a greater span of ∆*G*° (∆∆*G*° ~ 3 kcal/mol) than ∆*G*^‡^*_out_* (∆*G*^‡^*_out_* ~ 1 kcal/mol) values, along the series. On the basis of the transition state theory, ∆*G*^‡^*_out_* corresponds to the equilibrium between ([**1**–**2**] + [**solvent**]) and [**solvent**–**1**–**2**]^‡^ whereas ∆*G*° describes the balance between ([**1**–**2**] + [**solvent**]) and ([**1**–**solvent**] + [**2**]). Since the initial states are for both processes the same and comparable in energy (Figure 3D), the observed LFER in Figure 3B must indicate a greater span in the stability of [**1**–**solvent**] complexes than the corresponding transition states [**solvent**–**1**–**2**]^‡^ (Figure 4A)! To partly account for the observation, we note that the transition state for gated exchange must be an “open” form of the basket with (a) no intramolecular hydrogen bond(s) and (b) somewhat extended surface. Such transient species should “more easily” accommodate differently sized and shaped solvents, than [**1**–**solvent**] complex having a “closed” surface, to give rise to a more narrow distribution of ∆*G*^‡^*_out_* values.

The energy separation between [**solvent**–**1**–**2**]^‡^ and the corresponding ground states is rather large (>10 kcal/mol, Figure 4A) and in line with a unique structure of this fleeting complex lacking N-H-N intramolecular hydrogen bonds [34]. With the assistance of ^1^H, ^1^H–EXSY spectroscopy, we measured rate coefficients *k*_out_ characterizing the departure of **2** from [**1**–**2**] at various temperatures in *m*-xylene-d_10_ (Table S5). From the Eyring plot (Figure 4B), we determined the activation parameters ∆*H*^‡^ = 10 ± 1 kcal/mol and ∆*S*^‡^ = −21 ± 4 kcal/mol K characterizing the process [21]. The negative entropy of activation (∆*S*^‡^ < 0) is indeed in line with an associative mechanism of exchange, resembling classical S_N_2 reactions, with simultaneous (but not necessarily synchronous) egress of **2** and ingress of *m*-xylene-d_10_ (Figure 4C)! In spite of the postulated one-step mechanism (Figure 2D), the exchange process may also be a two-step process including rapid opening of the basket’s one gate (first step) followed by a slower swapping of the entrapped with the incoming compounds and the simultaneous opening of two additional gates (second step). It is indeed difficult to repudiate this particular mechanistic scenario, although we have not observed any intermediate either spectroscopically or kinetically (the saturation kinetics) [18] to suggest the two-step process [23]. In addition, en earlier study [26] indicated that for a guest populating the basket’s inner space to a greater extent (PC > 0.3, Table 1) the basket’s racemization necessitates simultaneous opening of all three gates.

**Figure 5:**
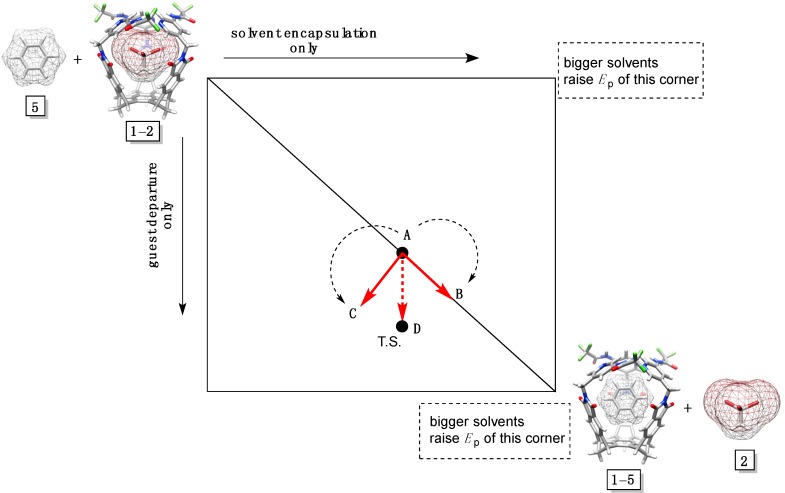
A More O’Ferrall-Jencks plot for solvents **3**–**6** (only **5** is shown) displacing 1,1,1-tribromoethane **2** in basket **1**.

To assess possible changes in the structure of the transition states for the postulated displacement mechanism (Figure 2D), we used the More O’Ferrall-Jencks diagram (Figure 5) [35]. Three reaction coordinates, as in classical nucleophilic substitutions [36], are included: (a) x-axis presents the solvent encapsulation without guest departure; (b) y-axis depicts the sole departure of guest (akin to S_N_1); and (c) the diagonal coordinate is a combination of the two pathways (akin to S_N_2). The movement of the transition state, with a change in the potential energy of the system (z-axis), proceeds along the diagonal reaction coordinate (Hammond effect) but, in accord with this approach, should also be balanced with a shift perpendicular to it (anti-Hammond effect) [36].

Bulkier solvent molecules form less stable complexes with basket **1** so that the top and bottom right corners are, in Figure 5, raised in the series **3**–**6**. Accordingly, the transition state shifts along AB and AC vectors to give rise to the AD vector. It follows that with an increase in the solvent size, the degree of solvent encapsulation stays constant while the magnitude of the guest departure increases. To put it in the language of classical organic chemistry, bulkier solvents enforce “S_N_1-like” mechanism of the supramolecular substitution: More sizeable mesitylene-d_12_ pushes the departing 1,1,1-tribromoethane **2** to a greater degree than benzene-d_6_ in the transition state [36,37]. The analysis is reasonable since bigger mesitylene-d_12_ provides less space than smaller benzene-d_6_ for departing compound **2** within the limited inner space of basket **1**. At last, the LFER in Figure 3A shows a linear dependence for the rate of the guest departure (log *k*_out_) as a function of the size (“nucleophilicity”) of solvents **3**–**6** thereby supporting the More O’Ferrall-Jencks analysis in Figure 5. In fact, this plot is analogous to Brönsted relationships [37] (log *k* = β_Nuc_·p*K*_a_ + n) that are used for characterizing the degree of covalent bond formation (β_Nuc_; which is the degree of solvent’s encapsulation in our case) in the transition state of substitution reactions as a function of nucleophilicity [35].

## 3. Experimental Section

All chemicals were purchased from commercial sources, and used as received unless stated otherwise. Solvents **3**, **4** and **6** were purchased from Cambridge Isotope Laboratories: benzene-d_6 _(D-99.5%), toluene-d_8_ (D-99.5%) and mesitylene-d_12_ (D-98%). *m*-Xylene-d_10_ (D-98%) was obtained from Sigma-Aldrich. All solvents (used in synthesis, see Scheme in supporting information) were dried prior to use according to standard literature protocols. Chromatography purifications were performed using silica gel 60 (Sorbent Technologies 40–75 μm, 200 × 400 mesh). Thin-layer chromatography (TLC) was performed on silica-gel plate w/UV254 (200 μm). ^1^H and ^13^C-NMR spectra were recorded (400 and 100 MHz, respectively), on a Bruker DPX-400 spectrometer unless otherwise noted. ^19^F NMR spectra were performed (376.54 MHz) on Bruker Biospin spectrometer with hexafluorobenzene (C_6_F_6_) as internal standard. All spectra were referenced using the solvent residual signal. The chemical shift values are expressed as δ (ppm) values. Coupling patterns are abbreviated as s (singlet), d (doublet), t (triplet), m (multiplet)*.* Temperatures for all NMR measurements were calibrated with neat methanol as a standard. High-resolution mass spectrometric measurements were completed on Bruker Micromass Q-TOF II spectrometer.

Compound **S2** (see Scheme S1 in SI): A solution of bromine (0.52 g, 3.3 mmol) in CCl_4_ (7 mL) was added to a solution of compound **S1** (1.0 g, 3.0 mmol) in CCl_4_ (20 mL) over 5 min at 77 °C. Following, the solvent was removed under reduced pressure to give brown oil. The oil was loaded on a thin pad of silica (SiO_2_), and then washed with dichloromethane (100 mL). The solvent was removed under reduced pressure to give compound **S2** as a light yellow solid (1.5 g, 99% yield; 2:1 mixture of two diastereomers). ^1^H NMR (400 MHz, CDCl_3_): δ (ppm) = 7.76–7.61 (m, 2 H, Ar*H*), 5.33 (d, *J* = 4.0 Hz, 0.3 H, C*H*), 4.45 (d, *J* = 2.8 Hz, 0.7 H, C*H*), 3.93 (s, 6 H, 2 × C*H*_3_), 2.83(m, 0.7 H, C*H_2_*), 2.72 (d, *J* = 8.1 Hz, 0.3 H, C*H_2_*), 2.38 (m, 0.3 H, C*H_2_*), 2.28 (m, 0.7 H, C*H_2_*). ^13^C NMR (100.62 MHz, CDCl_3_): δ (ppm) = 168.05, 168.01, 167.98, 167.68, 147.63, 146.94, 146.67, 145.67, 132.60, 131.50, 131.30, 131.27, 125.47, 125.14, 124.87, 122.05, 67.27, 66.90, 65.07, 63.69, 63.25, 63.11, 55.42, 52.94, 52.93, 52.88, 52.44, 47.23, 46.78. HRMS (ESI) *m/z* calcd for C_15_H_13_Br_3_O_4_Na [M+Na]^+^ 516.8256, found 516.8272.

Compound **S3** (see Scheme S1 in SI): A solution of potassium *tert*-butoxide (0.65 g, 1.3 mmol) in dry THF (20 mL) was added, by a syringe pump (3 h), to a stirred solution of compound **S2** (0.22 g, 2.0 mmol) in dry THF (100 mL) at 0 °C. After the addition was complete, the reaction mixture was stirred for additional 2 h at 0 °C. Following, the reaction mixture was quenched with cold acidic water (HCl aq), and the solvent evaporated under reduced pressure. The solid residue was partitioned between water (100 mL) and ethyl acetate (3 × 80 mL). Combined organic layers were dried over Na_2_SO_4_ and then evaporated under reduced pressure. The solid residue was purified by column chromatography (SiO_2_, hexane/ethyl acetate = 3:1) to yield compound **S3** as a white solid (0.43 g, 80% yield); m.p. 110–111 °C. ^1^H-NMR (400 MHz, CDCl_3_): δ (ppm) = 7.65 (s, 2 H, 2 × C*H*), 4.00 (m, 2 H, 2 × C*H*), 3.90 (s, 6 H, 2 × C*H*_3_), 2.79 (m, 1 H, C*H*_2_), 2.42 (m, 1 H, C*H*_2_). ^13^C NMR (100.62 MHz, CDCl_3_): δ (ppm) = 166.78, 150.38, 132.01, 128.90, 120.85, 65.69, 57.30, 51.40. HRMS (ESI) *m/z* calcd for C_15_H_12_Br_2_O_4_Na [M+Na]^+^ 438.8975, found 438.8972.

Compound **S4** (see Scheme S1 in SI): A solid mixture of Pd(OAc)_2_ (57 mg, 0.25 mmol), Ph_3_P (0.13 g, 0.51 mmol), Bu_4_NBr (1.7 g, 5.1 mmol), K_2_CO_3_ (3.5 g, 25.4 mmol) and 4Å molecular sieves (4.7 g) was added to a solution of dry dioxane (100 mL) under an atmosphere of argon. Compound **S3** (0.53 g, 1.3 mmol) was added and the reaction mixture stirred at 100 °C for additional 48 h. After, the reaction suspension was cooled to a room temperature and filtered with a filter paper. The solid residue was washed with ethyl acetate (~150 mL), and the organic solvent evaporated under reduced pressure to yield a crude solid residue. The residue was partitioned between water (100 mL) and ethyl acetate (3 × 80 mL). Combined organic layers were evaporated under vacuum, and the solid residue purified by column chromatography (SiO_2_, hexane/ethyl acetate = 2:1) to yield compound **S4** (59 mg, 20% yield); note that *anti* stereoisomer of **S4** was also isolated (60 mg, 20% yield). ^1^H-NMR (400 MHz, CDCl_3_): δ (ppm) = 7.43 (s, 6 H, 6 × C*H*), 4.41 (s, 6 H, 6 × C*H*), 3.78 (s, 18 H, 6 × C*H*_3_), 2.52 (m, 6 H, 3 × C*H*_2_). ^1^H-NMR of *anti* diastereomer of **S4** (400 MHz, CDCl_3_): δ (ppm) = 7.64 (s, 2 H, 2 × C*H*), 7.54 (s, 2 H, 2 × C*H*), 7.51 (s, 2 H, 2 × C*H*), 4.40 (s, 2 H, 2 × C*H*), 4.37, (s, 2 H, 2 × C*H*), 4.36 (s, 2 H, 2 × C*H*), 3.81–3.90 (s, 18 H, 2 × C*H*_3_), 2.48 (m, 2 H, C*H*_2_), 2.44 (m, 1 H, C*H*_2_), 2.37 (m, 2 H, C*H*_2_), 2.24 (m, 1 H, C*H*_2_).

The supplementary information includes: ^1^H-NMR Binding Studies, DOSY NMR/^1^H, ^1^H-EXSY spectroscopic measurements, variable temperature NMR measurements and ^19^F-NMR quantification of the solubility of basket **1**.

## 4. Conclusions 

A fundamental understanding of encapsulation processes mediated by dynamic hosts is important for controlling the outcome of chemical reactions [38], resolution of enantiomers by gating [29], and completing a precise delivery of molecules [39,40]. The results of our study contribute to such efforts. In particular, the experimental data suggest that the transition state corresponding to the rate-determining step of gated molecular encapsulations encompasses a conglomerate of the host and two molecules exchanging. In particular, the solvent molecule “pushes” a guest, trapped in the gated basket, to give rise to a transient structure containing both species within the concave surface of the unfolded basket. Importantly, bigger solvents are deduce to enforce “S_N_1-like” mechanism [41] of the exchange by advancing the degree of the guest departure.

## Supplementary Mterials

Supplementary materials can be accessed at: http://www.mdpi.com/1420-3049/19/9/14292/s1.

## Supplementary Files

Supplementary File 1
