# Supplementary material for: On the Nature of the Transition State Characterizing Gated Molecular Encapsulations
Source: Molecules. 2014 Sep 11;19(9):14292–303. doi: 10.3390/molecules190914292 (PMC6271723; doi:10.3390/molecules190914292)
Supplement: Supplementary File 1 [file molecules-19-14292-s001.pdf]

Supplementary Materials

S1. Synthesis

Basket **1** was synthesized by optimized procedure shown in the Scheme S1 below [1]. In particular, we discovered that palladium-catalyzed cyclotrimerization of dibromoolefin **S3** would give compound **S4** in 20% yield [1–3]. Compound **2** (Figure 1, main text) was obtained following an already published procedure [4].

Scheme S1. Optimized synthesis of basket **1**.

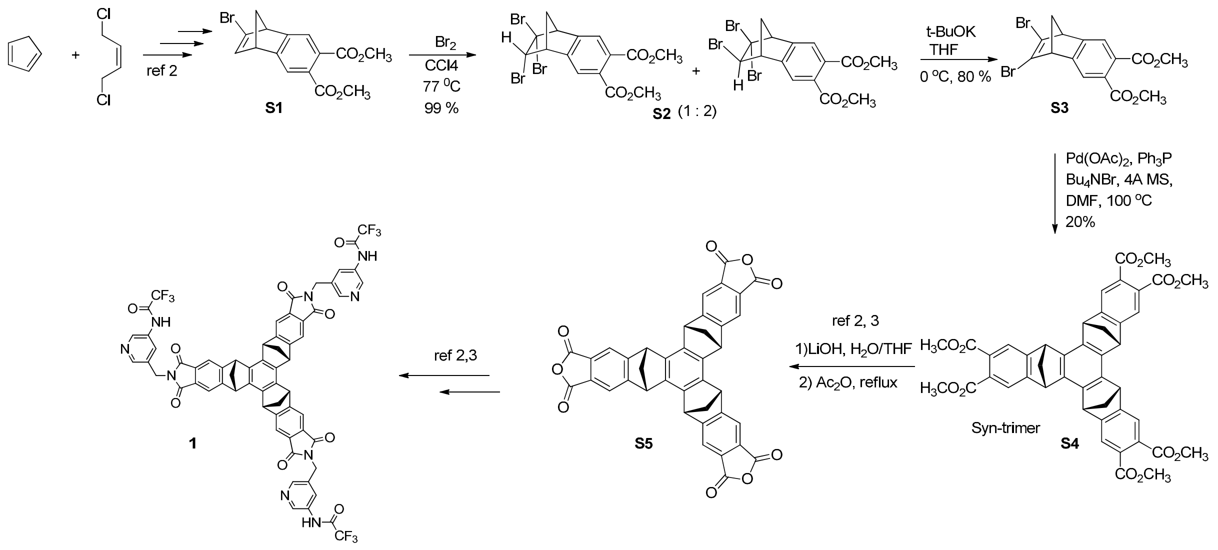

S2. Variable Temperature ^{1}H-NMR Spectroscopic Study of Basket 1

Figure S1. Variable temperature ^{1}H-NMR spectra (400 MHz) of basket **1** (1.1 mg) suspended in *m*-xylene-d_{10} (0.5 mL) at 298.0–348.0 K.

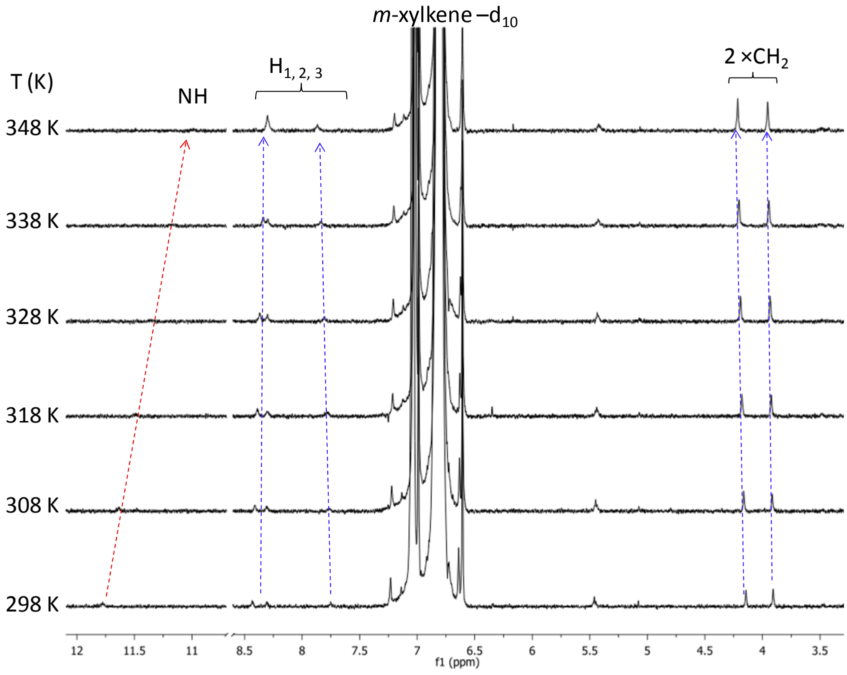

S3. ^{1}H-NMR Binding Studies

Since the rate of chemical exchange between basket **1** in its free [**1**] and complexed [**1**–**2**] forms was slow on the NMR time scale (300.1 K), in solvents **3**–**6**, we integrated N-H signals corresponding to **1** (in its free and complexed forms) to calculate binding constants K_a (M^{-1}). Figures S2–S5 depict ^{1}H-NMR spectra (400 MHz) of basket **1**, in solvents **3**–**6**, containing various quantities of guest **2**; note that a standard solution of guest **2** (see below) was used in all experiments.

Figure S2. ^{1}H-NMR spectra (400 MHz, 300.1 K) of basket **1** (0.54 mg) in benzene-d_6 (0.5 mL) obtained upon an incremental addition of a standard solution of CH_3CBr_3 **2** in benzene-d_6 (250 mM).

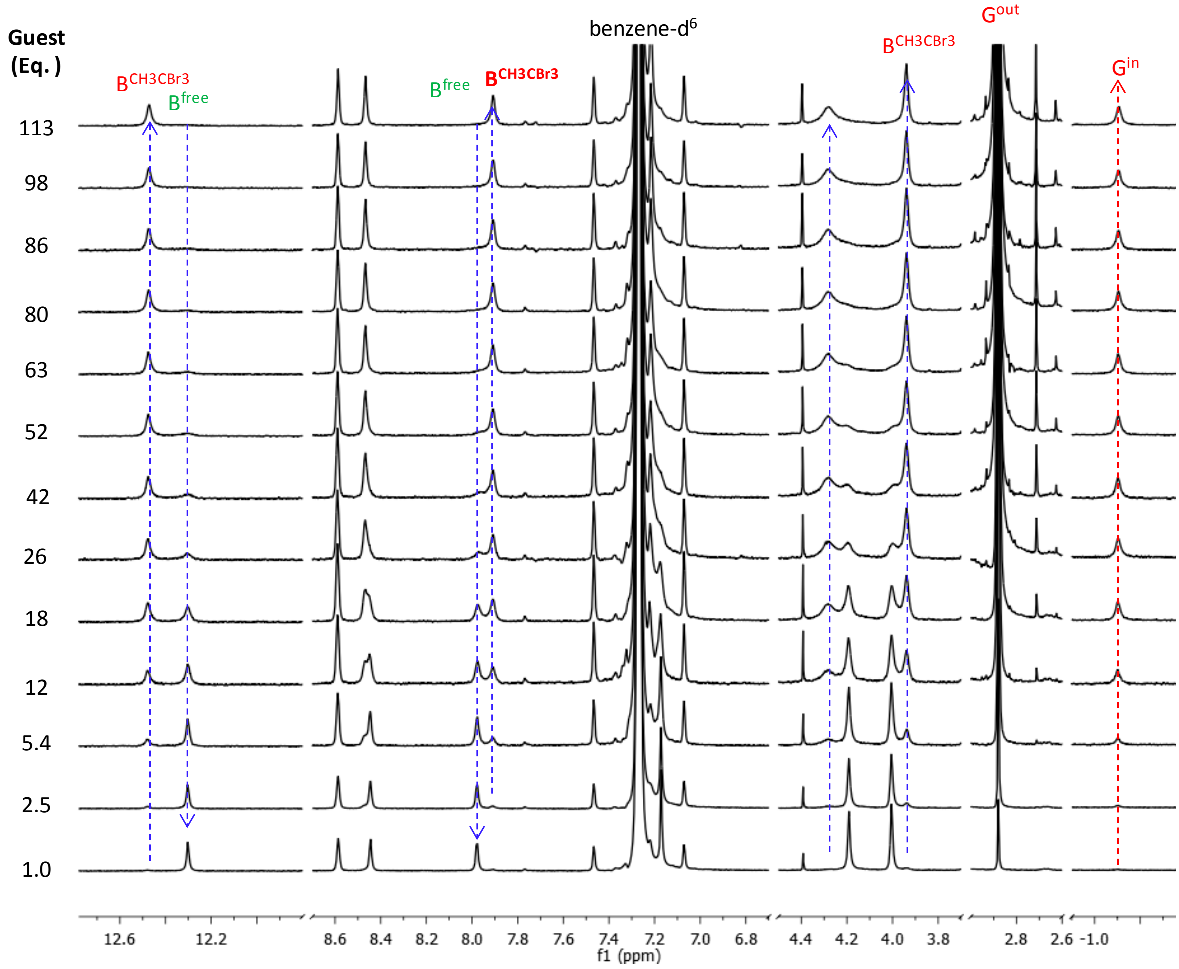

Figure S3. ^{1}H-NMR spectra (400 MHz, 300.1 K) of basket **1** (0.54 mg) in toluene-d_8 (0.5 mL) obtained upon an incremental addition of a standard solution of CH_3CBr_3 **2** in toluene-d_8 (112 mM).

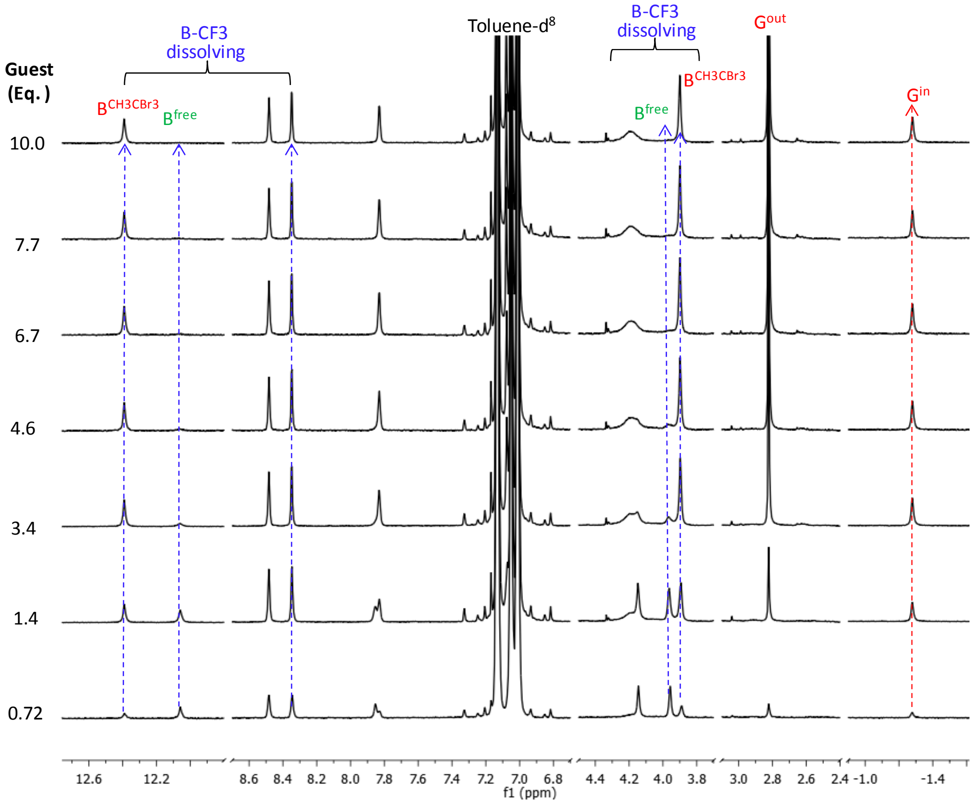

Figure S4. ^{1}H-NMR spectra (400 MHz, 300.1 K) of basket **1** (0.54 mg) in *m*-xylene-d_{10} (0.5 mL) obtained upon an incremental addition of a standard solution of CH_3CBr_3 **2** in *m*-xylene-d_{10} (250 mM).

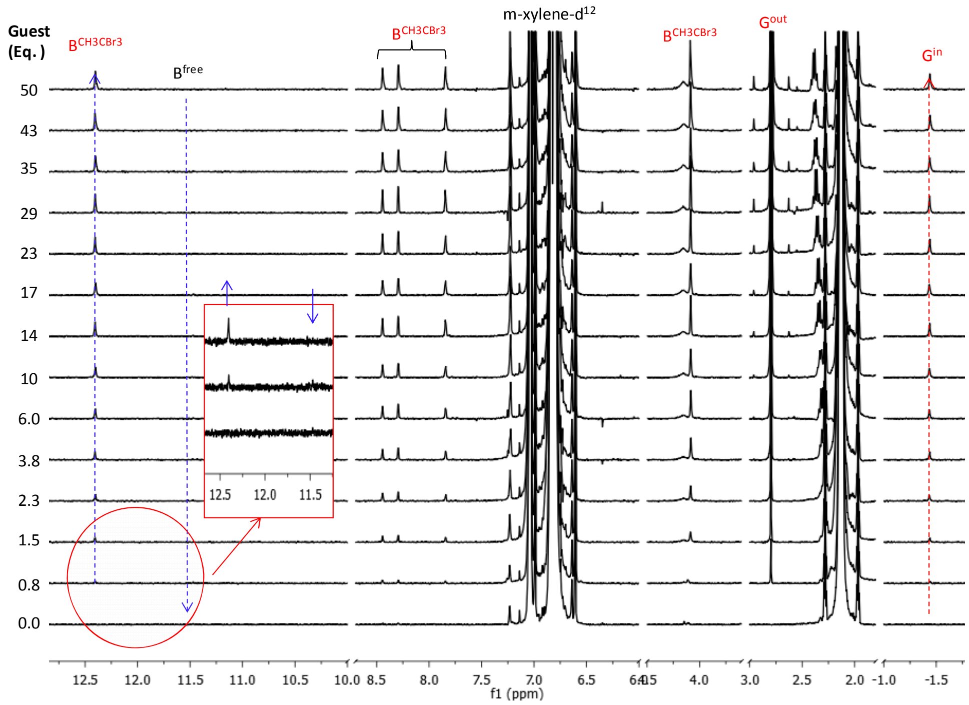

Figure. S5. ^{1}H-NMR spectra (400 MHz, 300.1 K) of basket **1** (0.54 mg) in mesitylene-d_{12} (0.5 mL) obtained upon an incremental addition of a standard solution of CH_3CBr_3 **2** in mesitylene-d_{12} (250 mM).

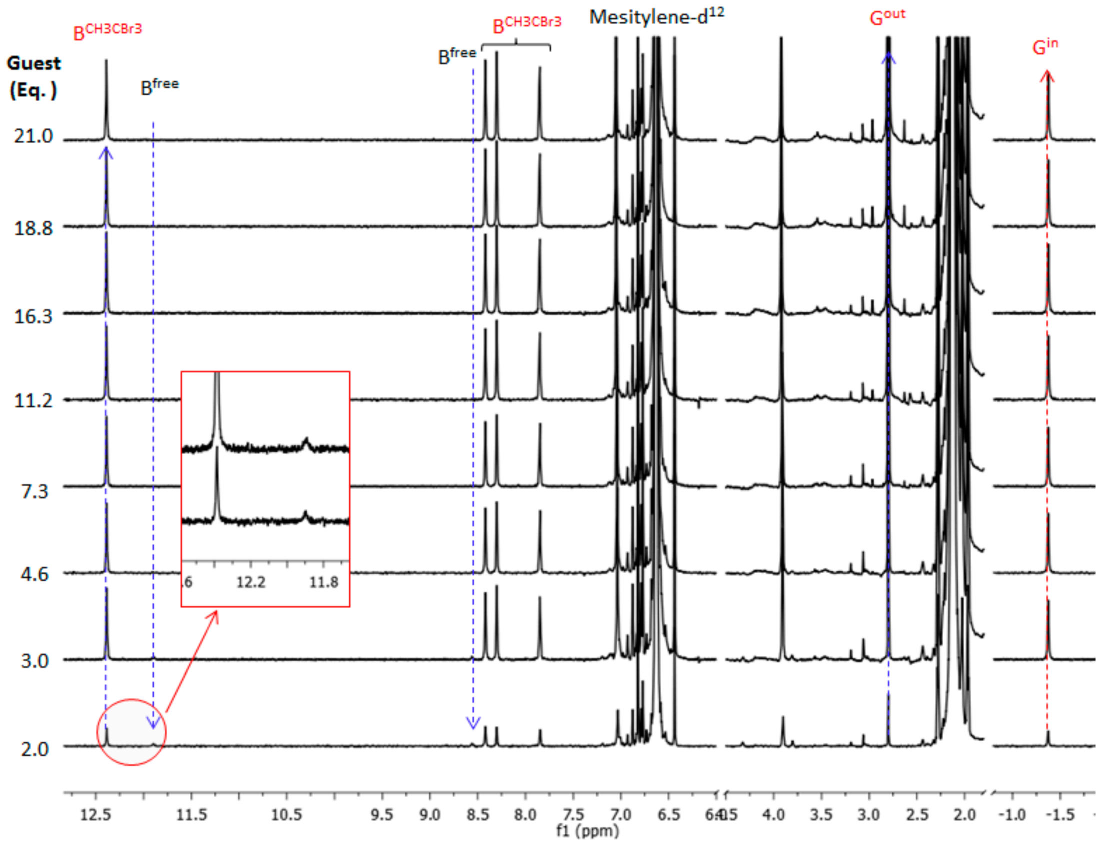

S4. DOSY NMR Experiments

Figure S6. ^{1}H DOSY NMR spectrum (500 MHz, 300.1 ± 0.5 K) of basket **1** (0.88 mM) and CH_3CBr_3 **2** (98.6 mM) in benzene-d_6 **3** (0.5 mL).

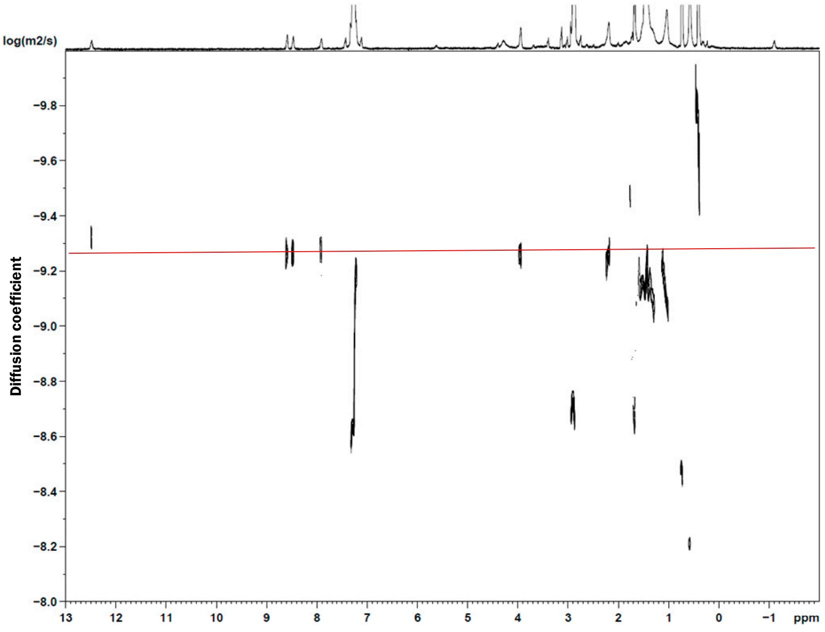

Figure S7. ^{1}H DOSY NMR spectrum (500 MHz, 300.1 ± 0.5 K) of basket **1** (0.88 mM) and CH_3CBr_3 **2** (10 mM) in toluene-d_8 **4** (0.5 mL).

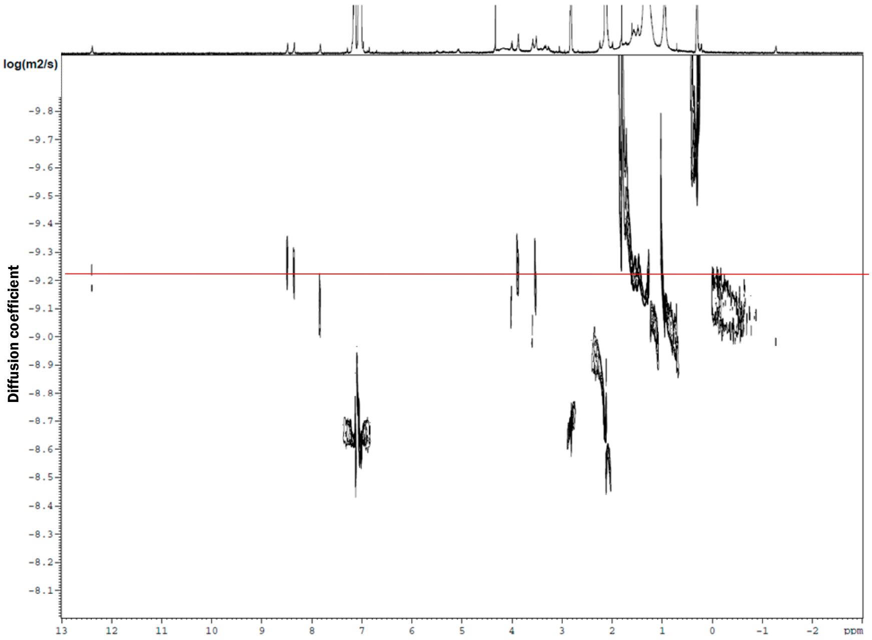

Figure S8. ^{1}H DOSY NMR spectrum (500 MHz, 300.1 ± 0.5 K) of basket **1** (0.88 mM) and CH_3CBr_3 **2** (19.4 mM) in *m*-xylene-d_{10} **5** (0.5 mL).

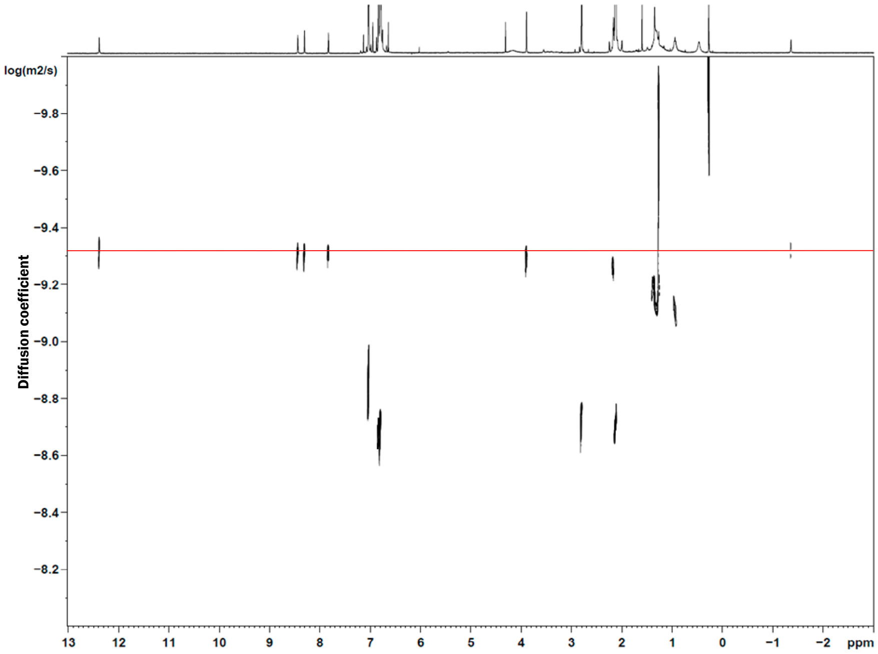

Figure S9. ^{1}H DOSY NMR spectrum (500 MHz, 300.1 ± 0.5 K) of basket **1** (0.88 mM) and CH_3CBr_3 **2** (16.7 mM) in mesitylene-d_{12} **6** (0.5 mL).

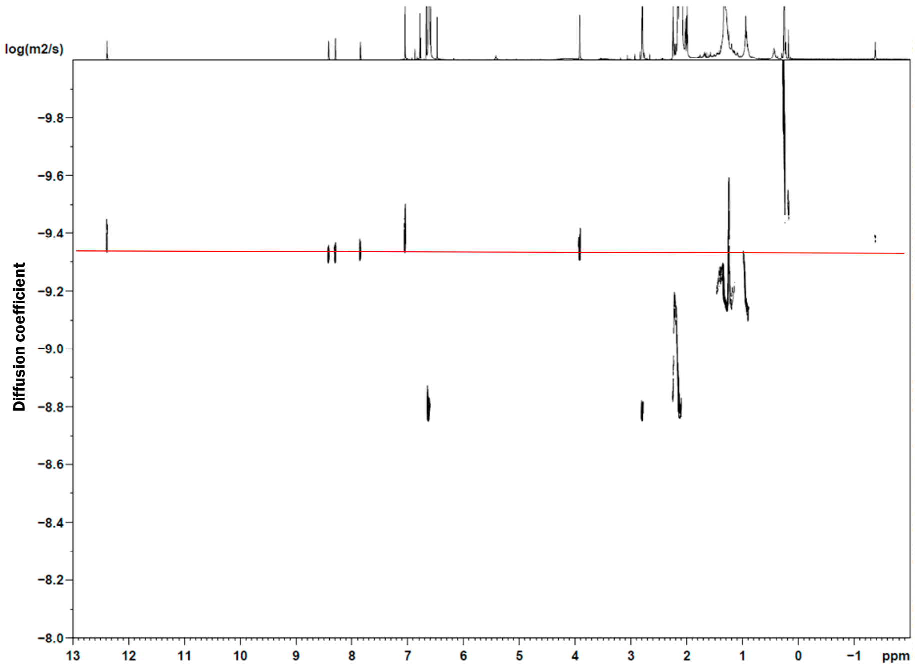

S5. ^{1}H, ^{1}H-EXSY Experiments

Sample Preparation: All deuterated solvents (**3**–**6**) were degassed by standard freeze-thaw procedure and then stored under nitrogen in a glove box. All solutions of **1** and **2** were, for EXSY experiments, prepared in J. Young NMR tubes purchased from Norell.

Procedure for 2-D ^{1}H EXSY Experiment: A solution of basket **1** and guest **2**, in solvent **3**–**6**, was kept at 300.0 ± 0.1 K inside the J. Young NMR tube for 30 min. The ^{1}H spin-lattice relaxation times (T_1) were, for free guest **2**, determined in each solvent by standard inversion-recovery pulse sequence. Following, a series of gradient EXSY experiments were run with a relaxation delay of 5*T_1 and mixing times (τ_m) of 0 ms and three others ranging from 250 ms to 450 ms such that the cross-peaks were clearly resolved. Each of the 128 F1 increments was the accumulation of at least 4 scans. The corresponding integrals were determined using MestReNova software from Mestrelab Research, after the phase and baseline corrections in both dimensions. The magnetization exchange rate constants (k_{out} *) were, at each mixing time τ_m, calculated using the EXSYCalc program (Mestrelab Research) [5]. The dissociation k_{out} rate constants were then obtained as: k_{out} = k_{out} *. All EXSY experiments were repeated twice. The mean value of k_{out} was reported with standard deviation as an experimental error (Table 1).

Table S1. Rate coefficients k_{out} (s^{-1}) (see Table below) were obtained from ^{1}H, ^{1}H-EXSY experiments (300.1 K) corresponding to CH_3CBr_3 **2** departing basket **1** (0.53 mg) in benzene-d_6 **3** (0.5 mL). We completed two sets of measurements with different quantity of CH_3CBr_3 at 300.1 ± 0.1 K.

Rate	*Eq. Guest*	**Mixing Time**		
		**300 ms**	**350 ms**	**400 ms**
k_{out} [s^{-1}]	17	/	19.137	17.996
			18.680	17.745
	22	21.357	21.969	16.544
		22.678	19.805	**17.795**

Figure S10. ^{1}H, ^{1}H-EXSY spectrum (400 MHz, 300.1 ± 0.1 K) of CH_3CBr_3 **2** (17 molar equivalents) exchanging to/from basket **1** (0.53 mg) in benzene-d_6 **3** (0.5 mL); note that for this particular experiment the mixing time τ_m was set to 400 ms.

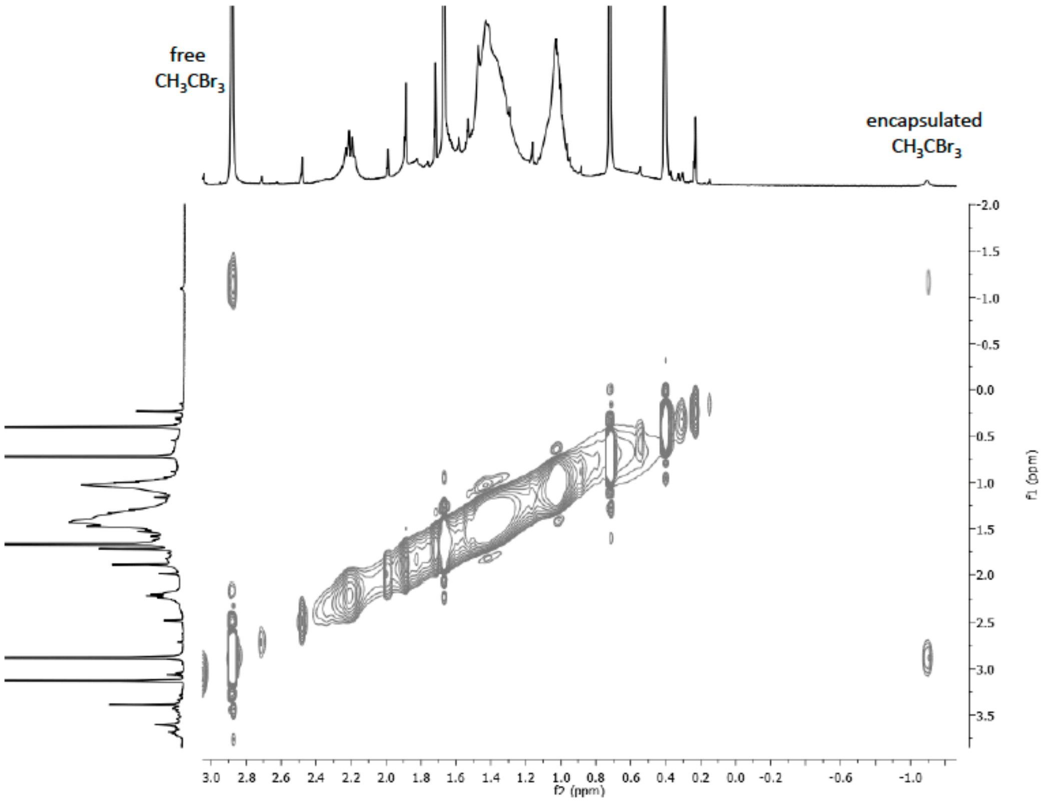

Table S2. Rate coefficients k_{out} (s^{-1}) (see Table below) were obtained from ^1H, ^1H-EXSY experiments (300.1 K) corresponding to CH_3CBr_3 **2** departing from basket **1** (0.60 mg) in toluene-d_8 **4** (0.5 mL). We completed three sets of measurements with different quantity of CH_3CBr_3 at 300.1 ± 0.1 K.

Rate	***Eq. Guest***	**Mixing Time**		
		**300 ms**	**350 ms**	**400 ms**
k_{out} [s^{-1}]	8.0	12.207	11.083	14.235
		13.688	8.672	10.559
	12	11.368	9.810	12.471
		10.848	10.284	9.983
	21	13.650	14.672	11.935
		12.933	11.990	12.565

Figure S11. ^1H, ^1H-EXSY spectrum (400 MHz, 300.1 ± 0.1 K) of CH_3CBr_3 **2** (8.0 molar equivalents) exchanging to/from basket **1** (0.60 mg) in toluene-d_8 **4** (0.5 mL); note that for this particular experiment the mixing time τ_m was set to 300 ms.

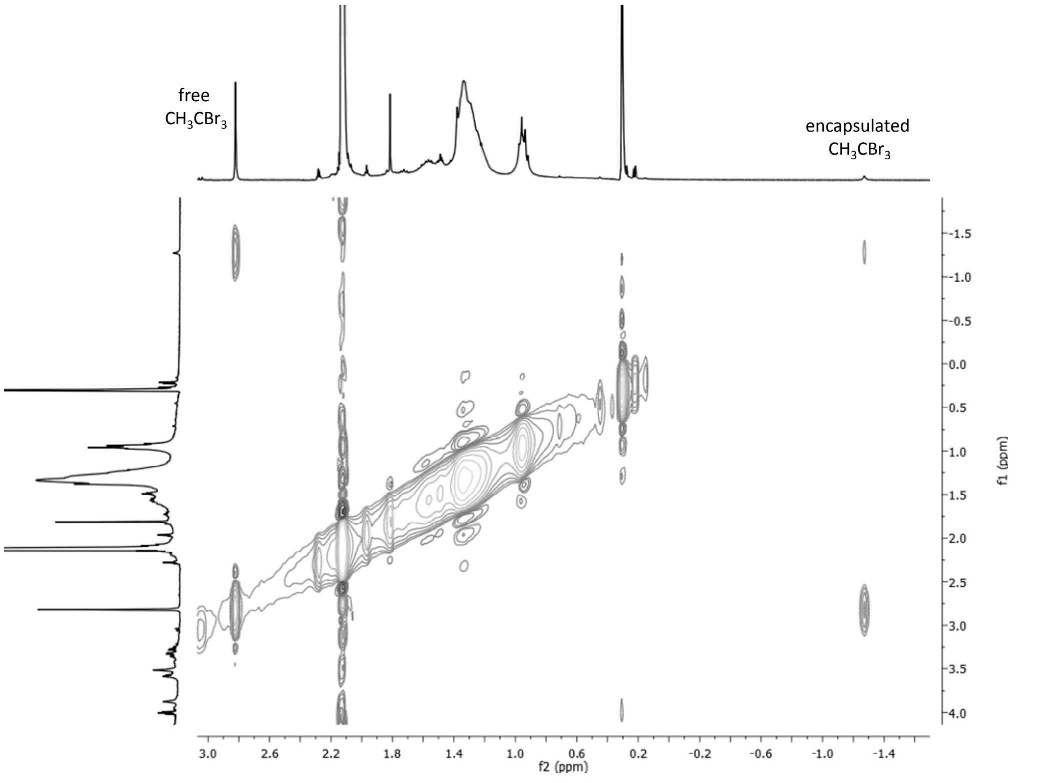

Table S3. Rate coefficients k_{out} (s^{-1}) (see Table below) were obtained from ^{1}H, ^{1}H-EXSY experiments (300.1 K) corresponding to CH_3CBr_3 **2** departing from basket **1** (1.1 mg) in *m*-xylene-d_{10} **5** (0.5 mL). We completed six sets of measurements with different quantity of CH_3CBr_3 at 300.1 ± 0.1 K.

Rate	*Eq. Guest*	**Mixing Time**		
		**300 ms**	**400 ms**	**450 ms**
k_{out} [s^{-1}]	3.0	9.219	8.026	7.135
		8.558	8.026	8.223
	5.6	7.377	6.439	5.899
		7.539	6.997	6.722
	10	8.758	8.717	6.687
		8.434	7.360	6.166
	15	11.803	9.888	9.202
		9.937	9.570	10.061
	20	10.404	10.040	9.090
		9.628		8.925
	22	7.535	7.691 7.625	7.832
		7.645		

Figure S12. ^{1}H, ^{1}H-EXSY spectrum (400 MHz, 300.1 ± 0.1 K) of CH_3CBr_3 **2** (10 molar equivalents) exchanging to/from basket **1** (1.1 mg) in *m*-xylene-d_{10} **5** (0.5 mL); note that for this particular experiment the mixing time τ_m was set to 400 ms.

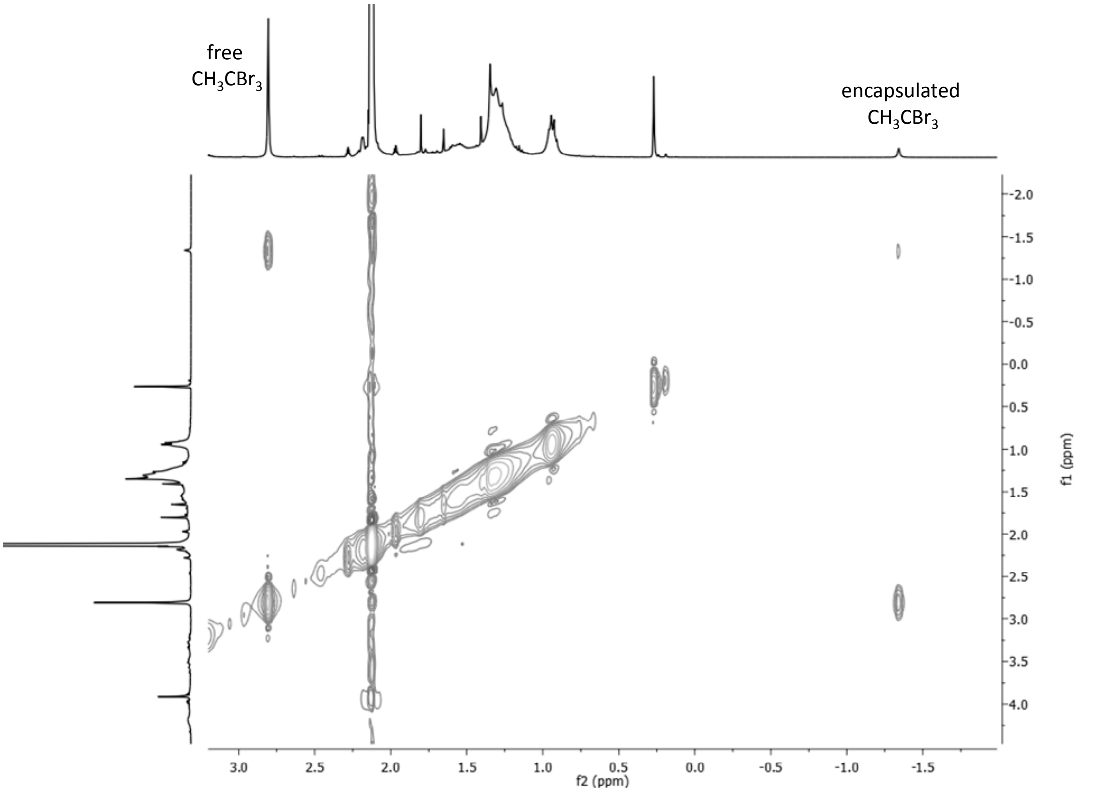

Table S4. Rate coefficients k_{out} (s^{-1}) (see Table below) were obtained from ^1H, ^1H EXSY experiments (300.1 K) corresponding to CH_3CBr_3 **2** departing from basket **1** (0.79 mg) in mesitylene-d_{12} **6** (0.5 mL). We completed four sets of measurements with different quantity of CH_3CBr_3 at 300.1 ± 0.1 K.

Rate	*Eq. Guest*	**Mixing Time**		
		**300 ms**	**350 ms**	**400 ms**
k_{out} [s^{-1}]	4.4	3.758	3.914	3.710
		3.304	3.923	3.710
	8.3	3.065	3.479	3.147
		3.104	3.052	3.097
	12	2.524	2.288	/
		2.583	2.197	
	20	4.512	4.657	4.314
		4.582	4.742	4.773

Figure S13. ^1H, ^1H-EXSY spectrum (400 MHz, 300.1 ± 0.1 K) of CH_3CBr_3 **2** (4.4 molar equivalents) exchanging to/from basket **1** (0.79 mg) in mesitylene-d_{12} **6** (0.5 mL); note that for this particular experiment the mixing time τ_m was set to 350 ms.

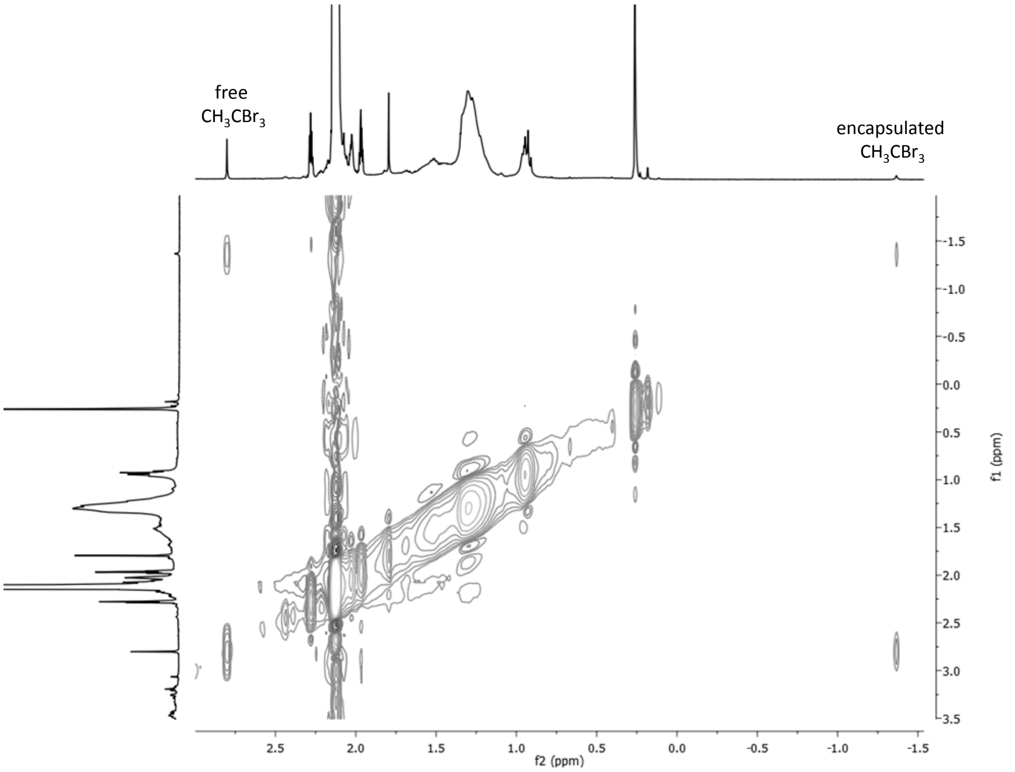

S6. Variable Temperature ^{1}H, ^{1}H EXSY-NMR Experiments

Table S5. ^{1}H,^{1}H-EXSY NMR experiments were completed at 306.3 ± 0.1, 311.5 ± 0.1, and 316.6 ± 0.1 K to obtain rate coefficients k_{out} (s^{-1}) for guest CH_3CBr_3 **2** (8.0 molar equivalents, 7.5 mM) departing basket **1** (0.54 mg) in *m*-xylene-d_{10} **5** (0.5 mL).

Rate	**T (K)**	**Mixing Time**		
		**300 ms**	**400 ms**	**450 ms**
k_{out} [s^{-1}]	306.3	9.697	9.124	9.361
		9.186	9.349	9.505
	311.5	15.247	13.531	12.456
		15.607	14.272	14.950
	316.6	17.114	16.602	17.246
		17.660	17.246	16.656

S7. Quantifying the Solubility of Basket 1

The solubility of basket **1** was measured in solvents **3**–**6** at 300.1 ± 0.1 K. First, we prepared a saturated solution of **1** in each solvent such that a precipitate always remained at the bottom of the container. Following, we added hexafluorobenzene (0.086 mmol) as an internal standard. ^{19}F NMR spectra (376.54 MHz; Bruker Biospin instrument) were recorded: 128 scans with 90° pulse sequence and relaxation delay τ_d = 20 s [6]. The solubility was calculated on the basis of the integration ratio of two ^{19}F signals: −75.81 ppm for basket **1** and −164.07 ppm for hexafluorobenzene reference (see below).

Figure S14. ^{19}F NMR spectra (376.54 MHz, 300.1 ± 0.1 K) of basket **1** in solvents **3**–**6** (from bottom to top) in the presence of C_6F_6 (0.15 mM) as an internal reference.

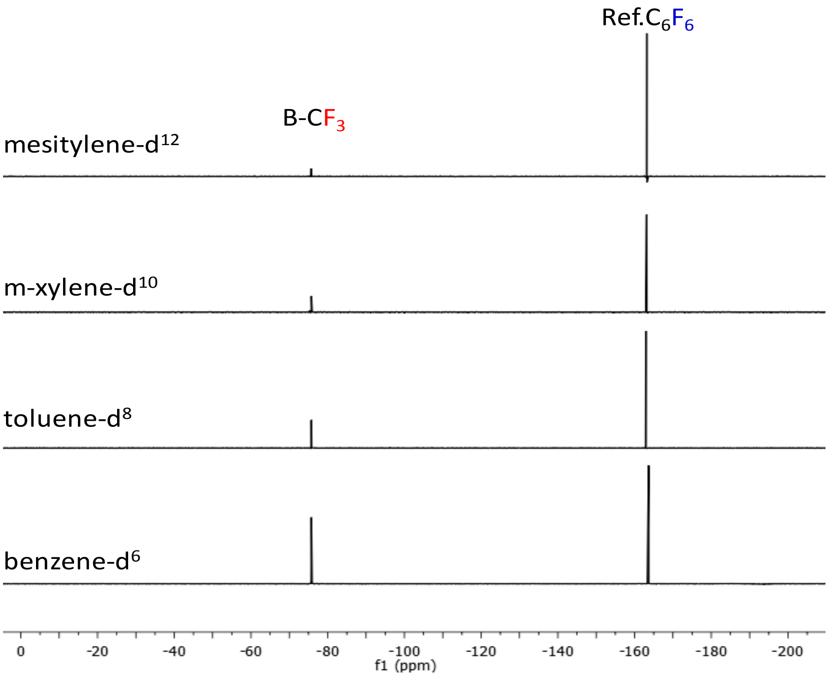

S8. Variable Temperature ^{1}H-NMR Studies of [1–2] Complex

Variable temperature ^{1}H-NMR Spectra were recorded for basket **1** (0.54 mg) and guest **2** (8.8–99.4 mM) in solvents **3**–**6**. The coalescence temperature of the signal(s) (~4.3 ppm) corresponding to CH_2 hydrogen nuclei (H_4, Figure 2) in **1** was found to be a function of the bulk solvent. That is to say, the rate of the racemization of basket **1** was found slower in bigger solvents (see Table S6).

Table S6. Coalescence temperature of basket **1** in solvents **3**–**6** obtained from variable temperature ^{1}H-NMR spectroscopy study.

Solvent	3	4	5	6
Coalescence T (K)	<282.6	283.7–287.8	287.8–291.9	299.1–300.1

Figure S15. Variable temperature ^{1}H-NMR spectra (400 MHz) of a solution of basket **1** (0.54 mg) containing CH_3CBr_3 **2** (99.4 mM) in benzene-d_6 **3** (0.5 mL).

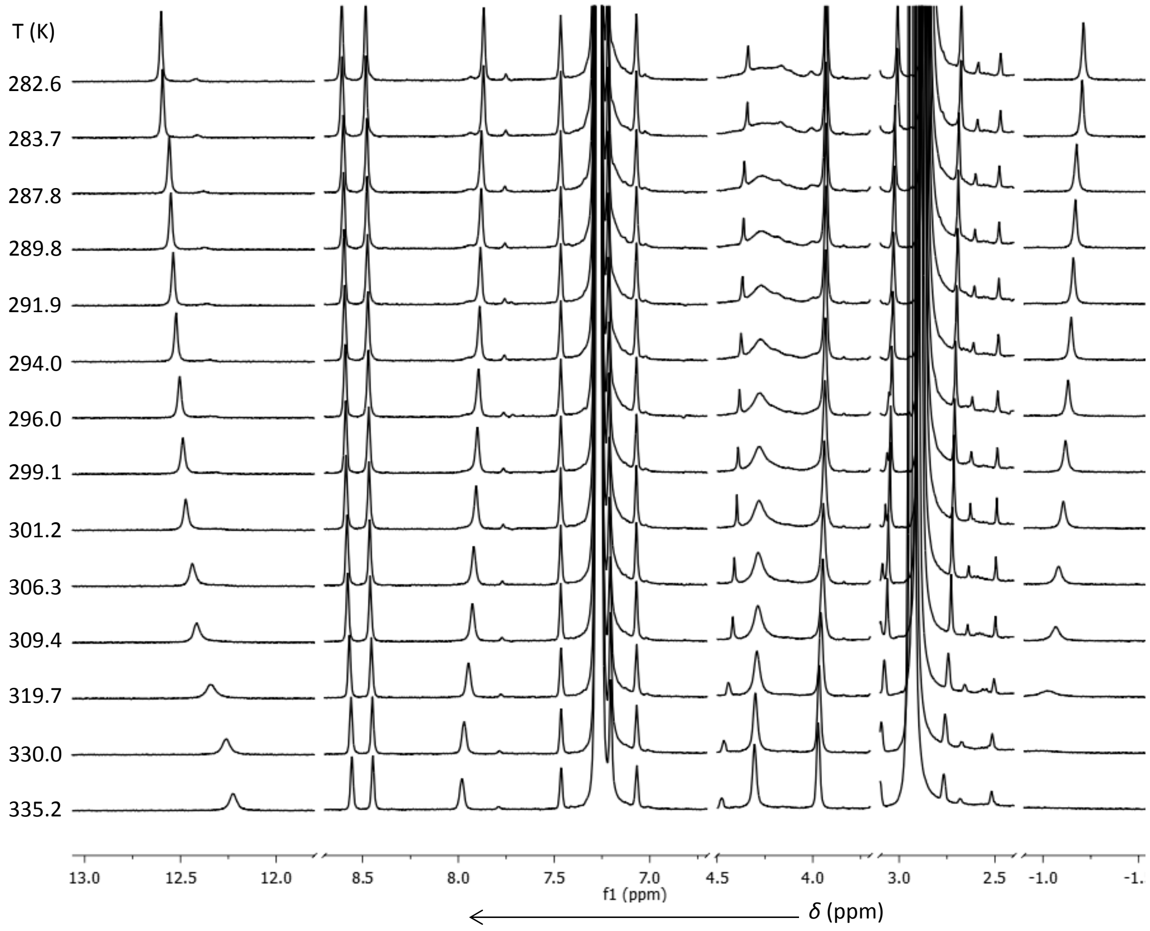

Figure S16. Variable temperature ^{1}H-NMR spectra (400 MHz) of a solution of basket **1** (0.54 mg) containing CH_3CBr_3 **2** (8.8 mM) in toluene-d_8 **4** (0.5 mL).

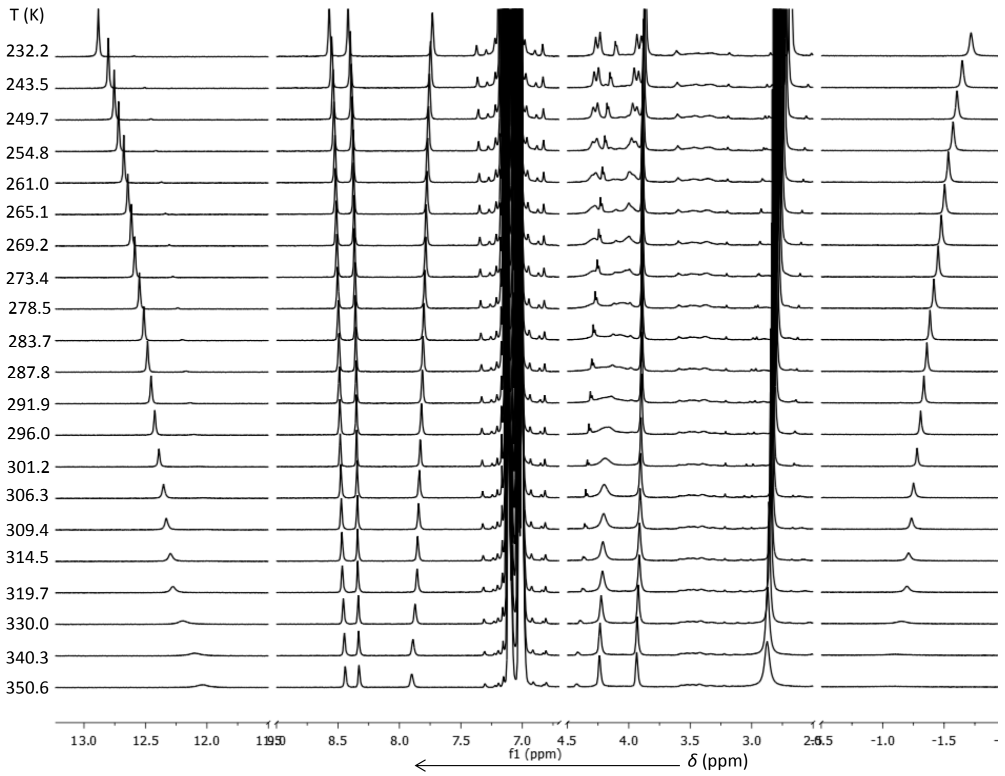

Figure S17. Variable temperature ^{1}H-NMR spectra (400 MHz) of a solution of basket **1** (0.54 mg) containing CH_3CBr_3 **2** (44 mM) in *m*-xylene-d_{10} **5** (0.5 mL).

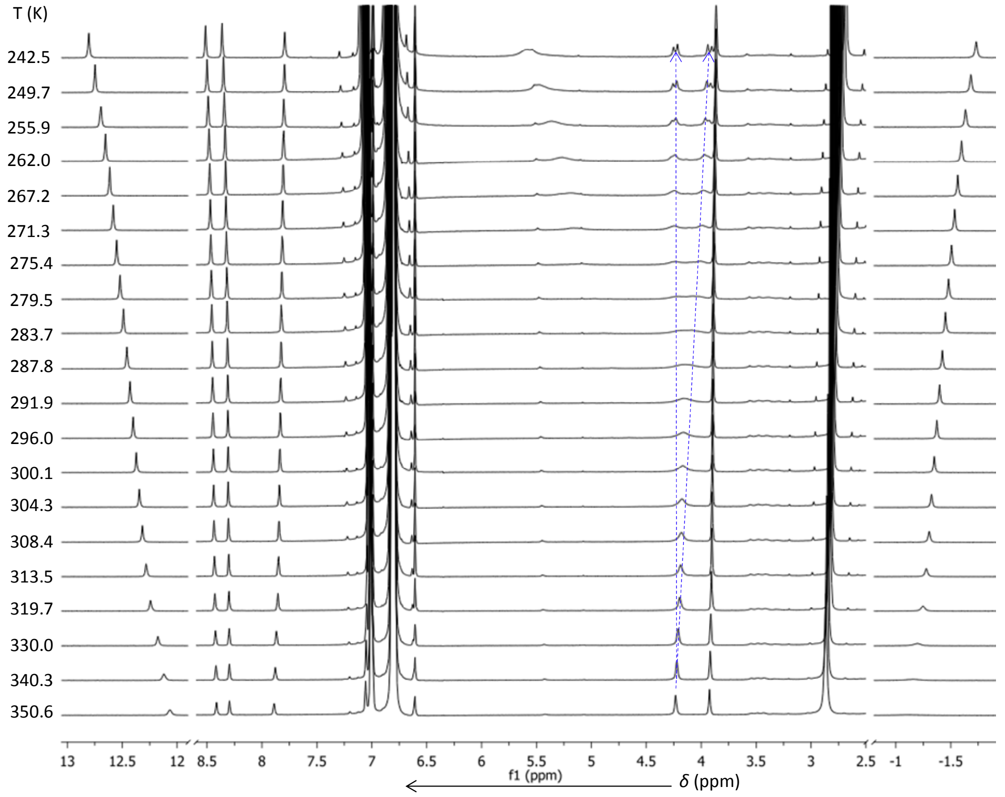

Figure S18. Variable temperature ^{1}H-NMR spectra (400 MHz) of a solution of basket **1** (0.54 mg) containing CH_3CBr_3 **2** (19 mM) in mesitylene-d_{12} **6** (0.5 mL).

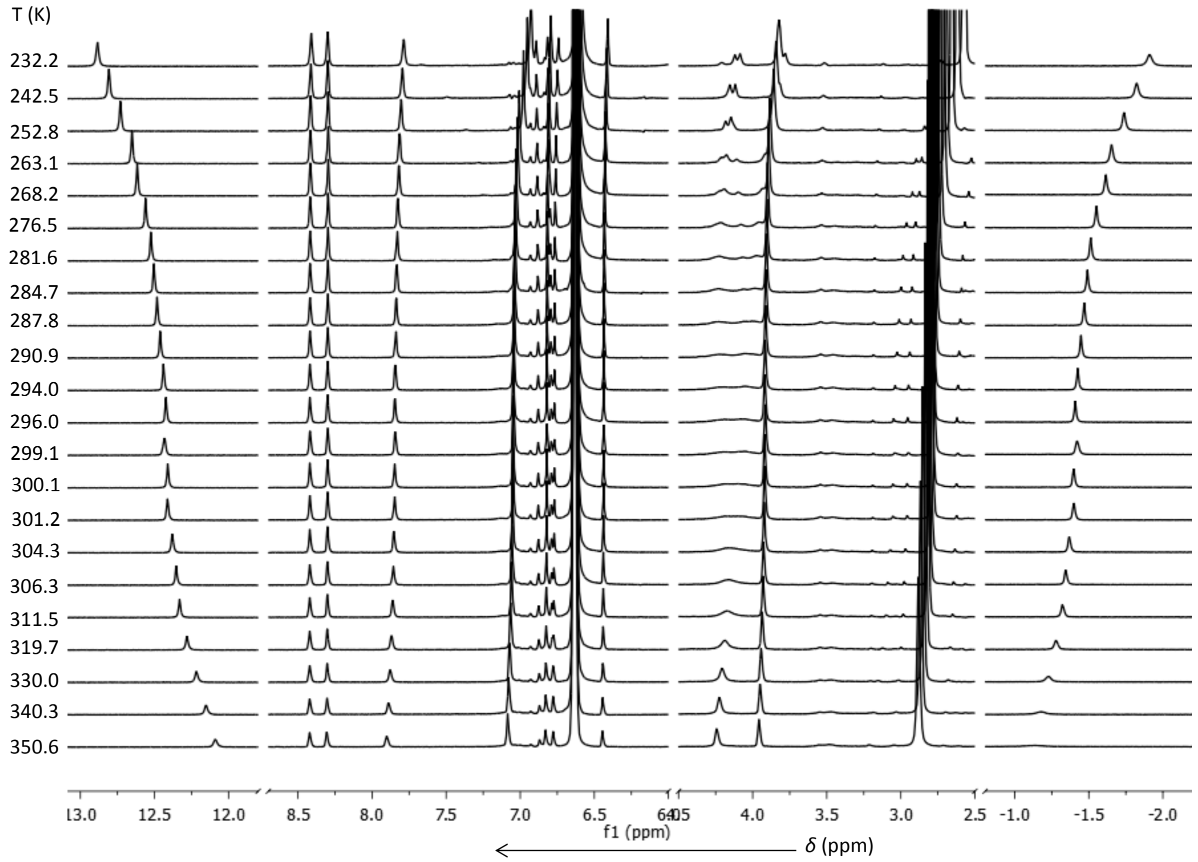

S9. ^{1}H- and ^{13}C-NMR Spectra of Compound S2 and S3

Figure S19. ^{1}H-NMR spectrum (400 MHz, $CDCl_3$) of compound **S2**.

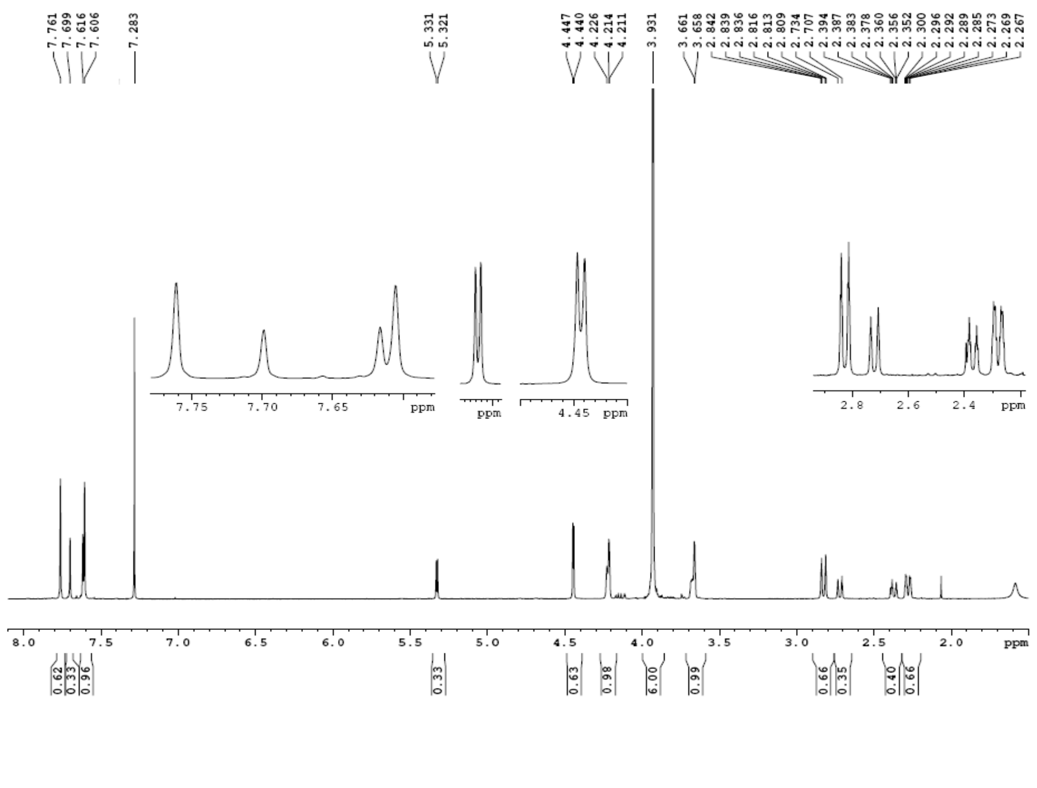

Figure S20. ^{13}C-NMR spectrum (100 MHz, $CDCl_3$) of compound **S2**.

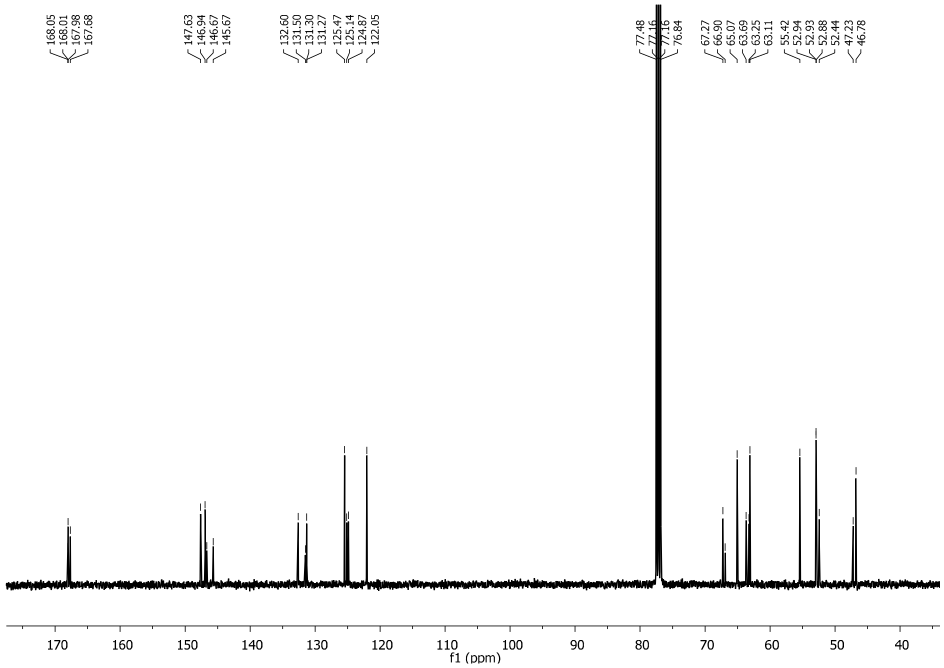

Figure S21. 2D HSQC NMR spectrum (100 MHz, $CDCl_3$) of compound **S2**.

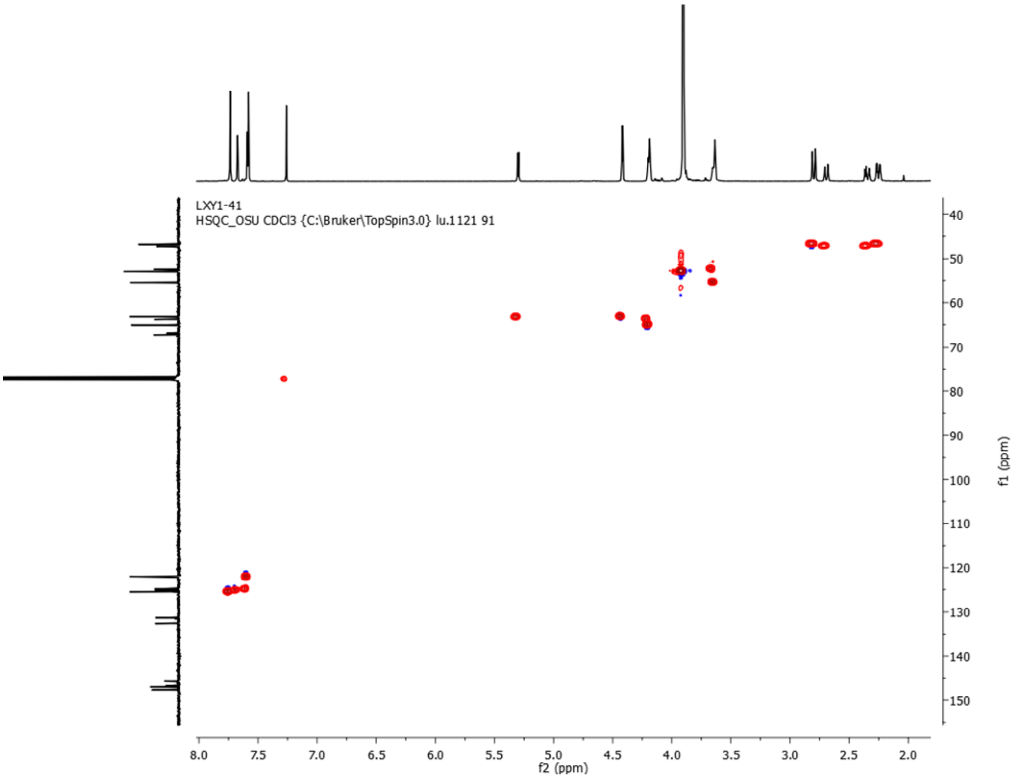

Figure 22. ^{1}H-NMR spectrum (400 MHz, $CDCl_3$) of compound **S3**.

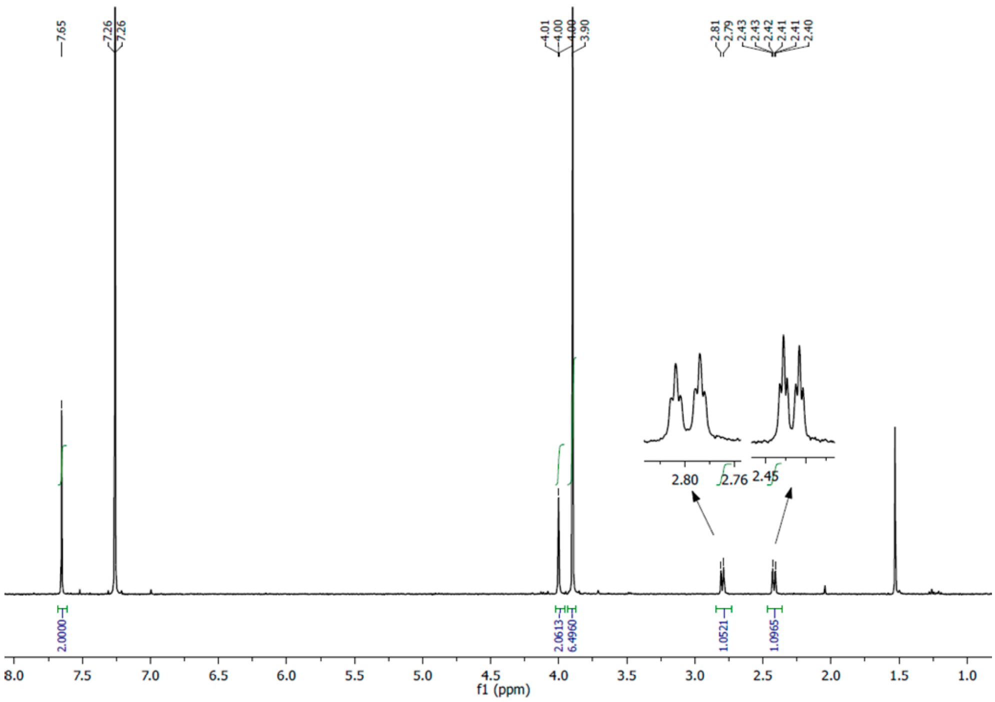

Figure S23. ^{13}C-NMR spectrum (100 MHz, $CDCl_3$) of compound **S3**.

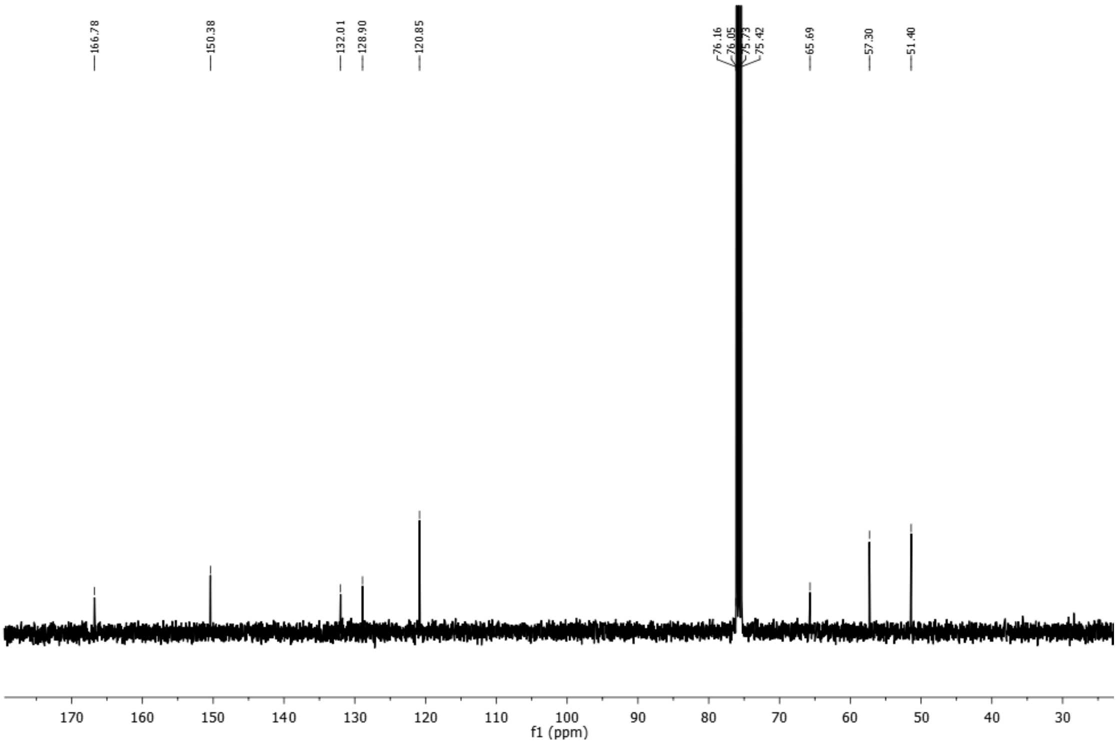

Figure S24. ¹H-NMR spectrum (400 MHz, $CDCl_3$) of *anti* diastereomer of compound **S4**.

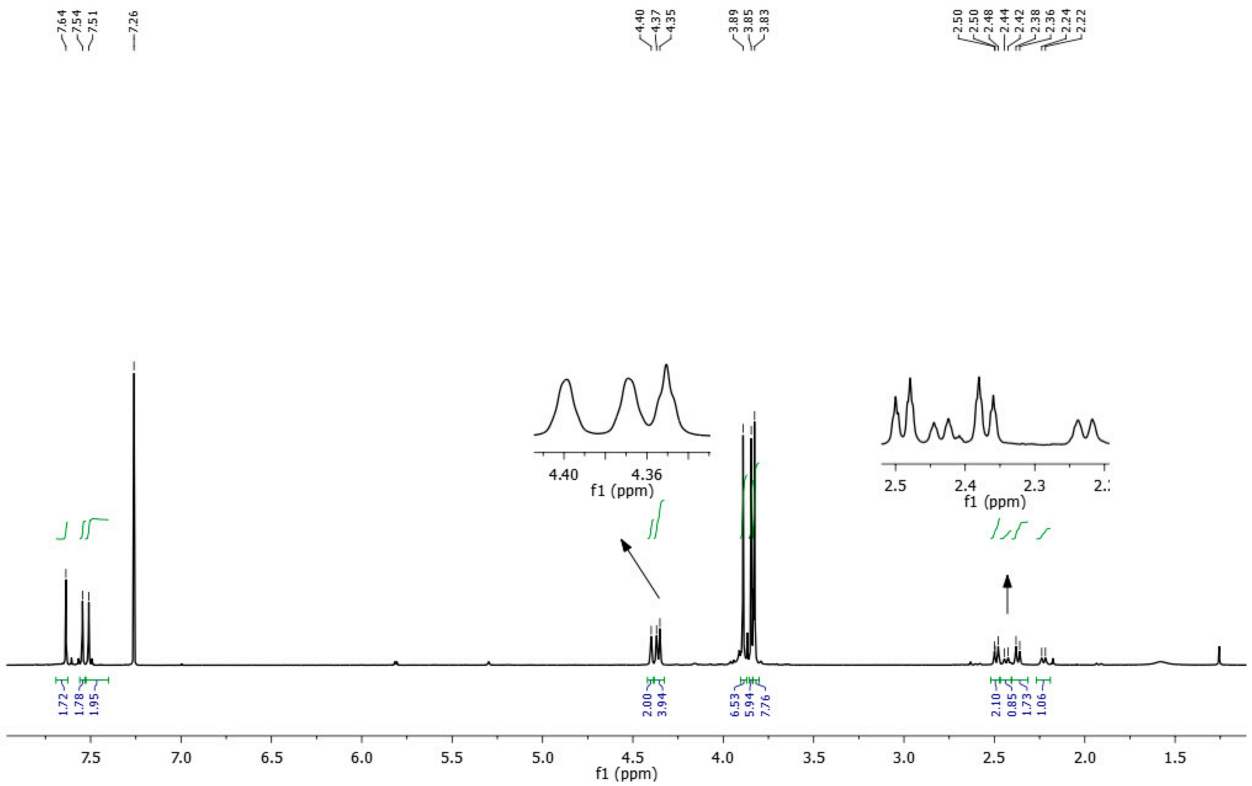

References

1. Maslak, V.; Yan, Z.; Xia, S.; Gallucci, J.; Hadad, C.M.; Badjić, J.D. Design, synthesis, and conformational dynamics of a gated molecular basket. *J. Am. Chem. Soc.* **2006**, *128*, 5887–5894.
2. Wang, B.; Rieth, S.; Badjic, J.D. Tuning the rate of molecular translocation. *J. Am. Chem. Soc.* **2009**, *131*, 7250–7252.
3. Hermann, K.; Sardini, S.; Ruan, Y.; Yoder, R.J.; Chakraborty, M.; Vyas, S.; Hadad, C.M.; Badjić, J.D. Method for the preparation of derivatives of heptiptycene: Toward dual-cavity baskets. *J. Org. Chem.* **2013**, *78*, 2984–2991.
4. Rieth, S.; Bao, X.; Wang, B.; Hadad, C.M.; Badjic, J.D. Gated molecular recognition and dynamic discrimination of guests. *J. Am. Chem. Soc.* **2010**, *132*, 773–776.
5. Zaolnai, Z.; Juranic, N.; Vikic-Topic, D.; Macura, S. Quantitative determination of magnetization exchange rate constants from a series of Two-Dimensional Exchange NMR spectra. *J. Chem. Inf. Comput. Sci.* **2000**, *40*, 611–621.
6. Delpuecha, J.-J.; Hamza, M.A.; Serratrice, G.; Stébé, M.-J. Fluorocarbons as oxygen carriers. I. An NMR study of oxygen solutions in hexafluorobenzene. *J. Chem. Phys.* **1979**, *70*, 2680–2687.
